# Impact of interventions to reduce nosocomial transmission of SARS-CoV-2 in English NHS Trusts: a computational modelling study

**DOI:** 10.1186/s12879-024-09330-z

**Published:** 2024-05-07

**Authors:** Stephanie Evans, James Stimson, Diane Pople, Peter J White, Mark H Wilcox, Julie V Robotham

**Affiliations:** 1 Fungal, HCAI, AMU & Sepsis Division, UK Health Security Agency, London, AMR UK; 2 Statistics, Modelling and Economics, UK Health Security Agency, London, UK; 3 MRC Centre for Global Infectious Disease Analysis, Imperial College London, London, UK; 4 NIHR Health Protection Research Unit in Modelling and Health Economics at Imperial College London in partnership with the UK Health Security Agency and London School of Hygiene and Tropical Medicine, London, UK; 5 Healthcare-Associated Infections Research Group, Leeds Institute of Medical Research, University of Leeds, Leeds, LS1 9JT UK; 6 Microbiology, Leeds Teaching Hospitals, Leeds, UK; 7 NIHR Health Protection Research Unit in Healthcare Associated Infections and Antimicrobial Resistance at University of Oxford in partnership with the, UK Health Security Agency, Oxford, UK

**Keywords:** Infection prevention and control, IPC, Healthcare-associated infection, Masking, Individual-based model, Agent-based model

## Abstract

**Background:**

Prior to September 2021, 55,000–90,000 hospital inpatients in England were identified as having a potentially nosocomial SARS-CoV-2 infection. This includes cases that were likely missed due to pauci- or asymptomatic infection. Further, high numbers of healthcare workers (HCWs) are thought to have been infected, and there is evidence that some of these cases may also have been nosocomially linked, with both HCW to HCW and patient to HCW transmission being reported. From the start of the SARS-CoV-2 pandemic interventions in hospitals such as testing patients on admission and universal mask wearing were introduced to stop spread within and between patient and HCW populations, the effectiveness of which are largely unknown.

**Materials/methods:**

Using an individual-based model of within-hospital transmission, we estimated the contribution of individual interventions (together and in combination) to the effectiveness of the overall package of interventions implemented in English hospitals during the COVID-19 pandemic. A panel of experts in infection prevention and control informed intervention choice and helped ensure the model reflected implementation in practice. Model parameters and associated uncertainty were derived using national and local data, literature review and formal elicitation of expert opinion. We simulated scenarios to explore how many nosocomial infections might have been seen in patients and HCWs if interventions had not been implemented. We simulated the time period from March-2020 to July-2022 encompassing different strains and multiple doses of vaccination.

**Results:**

Modelling results suggest that in a scenario without inpatient testing, infection prevention and control measures, and reductions in occupancy and visitors, the number of patients developing a nosocomial SARS-CoV-2 infection could have been twice as high over the course of the pandemic, and over 600,000 HCWs could have been infected in the first wave alone. Isolation of symptomatic HCWs and universal masking by HCWs were the most effective interventions for preventing infections in both patient and HCW populations. Model findings suggest that collectively the interventions introduced over the SARS-CoV-2 pandemic in England averted 400,000 (240,000 – 500,000) infections in inpatients and 410,000 (370,000 – 450,000) HCW infections.

**Conclusions:**

Interventions to reduce the spread of nosocomial infections have varying impact, but the package of interventions implemented in England significantly reduced nosocomial transmission to both patients and HCWs over the SARS-CoV-2 pandemic.

**Supplementary Information:**

The online version contains supplementary material available at 10.1186/s12879-024-09330-z.

## Introduction

Over the course of the COVID-19 pandemic in England, there has been evidence of nosocomial SARS-CoV-2 transmission to both patients and healthcare workers (HCWs) [1–4], with 0.5–1.25% of susceptible inpatients (55,000–90,000 patients) identified as having developed a nosocomial infection between 01-March-2020 and 01-Sept-2021 [5, 6]. Several interventions have been introduced in hospitals to reduce the transmission rate of SARS-CoV-2 including regular testing of patients and HCWs, increased hand-hygiene, and HCWs wearing masks/face coverings universally (i.e., around both patients and HCWs), in addition to hospital system changes such as reductions in occupancy [7, 8]. It is difficult to assess the effectiveness of individual interventions through data-driven approaches alone because several measures were implemented in quick succession. The emergence of vaccines and new variants have also altered nosocomial transmission rates over time, providing further uncertainty [5]. Computational modelling can help address these issues by providing a framework where individual interventions can be removed/reversed individually or in combination, counterfactual simulations can be executed, and the effectiveness of interventions assessed [9]. Using a previously developed computational model [10] we present an estimate of the impact of interventions in place during the pandemic, provide a counterfactual analysis of what might have happened had they not been implemented, and predict which combinations of interventions have the highest impact on transmission to both patients and HCWs.

We estimated the impact of eight hospital interventions/changes on nosocomial infections in both patients and HCW by 1) combining evidence from the literature on the efficacy/effectiveness of individual measures for reducing the spread of respiratory viral infections in hospitals and the community; 2) modifying model parameters to reflect scenarios in which the interventions were ‘reversed’ (both individually and collectively), e.g., through modifying the probability of transmission from inpatients and HCWs; and 3) modelling counterfactual scenarios in which interventions were ‘reversed’, i.e., simulating the hypothetical scenario in which the interventions/changes had not been implemented, to estimate how much virus transmission and resulting infections they averted individually and when combined.

## Methods

### Model development and calibration

We have extended an existing individual-based model (IBM) of nosocomial transmission within and between patient and HCW populations [9, 10]. The model simulates transmission through different routes: i) patient-to-patient transmission between those sharing a bay, ii) patient-to-patient transmission between patients residing on the same ward but not necessarily in the same bay, representing transmission through, for example, fomites, shared facilities, or transient asymptomatic carriage by HCWs, among others, iii) patient-to-HCW transmission, iv) HCW-to-patient transmission, v) HCW-to-HCW while present on the same ward, and vi) HCW-to-HCW anywhere in the hospital. Indirect transmission is captured implicitly in the model as a result of transmission occurring on wards between any HCWs that have shared a space within a 4 h time step (and so in practice may not have occupied that space concurrently) and through the indirect transmission route whereby HCWs anywhere in the hospital may infect other staff with no explicit requirement for them to physically share a space at any time. Infected patient cases are imported from the community at a rate calculated from observed hospital admissions in the NHSE Situation Report data [6]. HCWs can become infected in the community when they are outside of the hospital according to the predicted community prevalence on that date, where the prevalence was calculated from the Cambridge Real-Time Model [11]. Under the baseline scenario, the IBM is parameterised using multiple national datasets and values from the literature and is calibrated to reproduce the transmission dynamics of SARS-CoV-2 among healthcare workers (HCWs) and patients in an average English hospital. A full model description and details of the calibration procedure are described in Supplementary File 1.

### Counterfactual modelling of ‘reversal’ of interventions

Counterfactual scenarios were simulated in which eight interventions were reversed (individually and collectively) and compared to a baseline where interventions were in place, with scenarios as follows: 1) Baseline – transmission and interventions simulated to reflect observed numbers of infections in the data; 2) removal of lateral flow device tests (LFDs) for HCWs that were introduced in November 2020; 3) reversal of improvements in hand-hygiene from the start of the pandemic; 4) reversal of testing and cohorting of symptomatic patients throughout the simulation period; 5) reversal of reduced occupancy, instead with occupancy remaining at 2019 levels March to May 2020; 6) reversal of suspension of visitors to hospital patients (March to October 2020); 7) removal of universal mask wearing by HCWs (i.e., HCWs wear masks around patients but not around other HCWs) from June 2020; 8) removal of mask wearing by HCWs when treating patients (i.e., HCWs do not wear masks around patients or other HCWs) throughout simulation period; 9) isolation of symptomatic HCWs removed throughout simulation period, 10) reversal of all interventions in 2–9 collectively. The reversal of interventions was parameterised through literature searches and expert elicitation where there was a paucity of evidence (Table 1, Supplementary File 2). A supplementary analysis was also performed exploring a further set of counterfactual scenarios where interventions were in place from the start of the pandemic instead of being introduced at different times as in reality.

**Table 1:** Modelling reversal of interventions

*Scenario*	*Date of implementation*	*Impact of reversal*	*How is reversing this intervention modelled?*
LFD testing for HCWs	16-Nov-2020 [12]	All asymptomatic staff continue to work (and interact with patients and other HCWs)	Twice weekly HCW testing with 70% compliance is removed. HCWs still test when symptomatic. The probability of detection is estimated based on time from infection as described in the literature [13]
Hand-hygiene	Entire pandemic	Transmission rates increase across all pathways (patient-to-patient, patient-to-HCW, HCW-to-HCW), both within and between bays	This intervention was parameterised only using expert elicitation since there was a paucity of evidence in the literature (Supplementary File 2). New parameter values were sampled from the joint distribution of each expert’s opinion
Patient testing and cohorting	24-April-2020 (admissions testing) [7] 24-June-2020 (repeat testing) [14]	The number of SARS-CoV-2 patients (both community and nosocomial) sharing wards or bays with susceptible admissions is increased as asymptomatic and presymptomatic cases are cohorted with susceptible patients	When testing and cohorting of patients was removed, patients were assigned a ward/bed/bay at random regardless of their SARS-CoV-2 status or symptoms
Occupancy	Gradual throughout pandemic [15]	Number of patients in hospital is maintained at fixed (higher) level	Occupancy level was reduced in March 2020, gradually increased back to pre-pandemic levels by the end of 2020, and remained at the higher level until the end of the study. To remove this intervention occupancy is increased to 2019 levels throughout
Visitors	03-March-2020 to 15-Oct-2020 [16]	Allowing visitors at pre-pandemic levels adds an additional risk of transmission to patients at a rate based on the community infection rates	This intervention was parameterised using the distributions from the expert elicitation (Supplementary File 2) for the number of visitors per patient per day. The probability a visitor was infected was set to the community prevalence level and the transmission probability was estimated based on the length of the visitation [17] (elicited from experts)
Masking (Universal)	15-June-2020 [18]	When the universal masking intervention is removed, the transmission rate between HCWs on a ward is increased, but transmission to HCWs from patients (and to patients from HCWs) is unchanged	The effectiveness of masking on transmission was randomly drawn from the 95% confidence interval from a meta-analysis of SARS-CoV-2 specific studies available on general masking (not specifically FFP3 or FRSM) in the literature (0.51 [0.28–0.95], Supplementary File 2). These values fell into the ranges identified by the expert elicitation exercise that also identified compliance levels of around 70%. As the compliance level in these studies was not recorded, we assume that the range identified by the meta-analysis covers the compliance and efficacy possibilities. Masking was reversed by increasing relevant transmission probabilities by this factor
Masking (around patients)	06-March-2020 [19]	When masking around patients is removed it is in addition to universal masking being removed and therefore, transmission rates are increased between patients and HCWs and between HCWs and other HCWs on the ward	
Isolation by HCWs	Entire pandemic	All infected staff continue to work unless they receive a positive LFD test (and therefore continue to interact with patients and other HCWs)	This is a scenario where symptoms in HCWs are sufficiently mild that they continue to work and are only tested through routine asymptomatic testing

After obtaining data for parameterising the reversal of interventions, new parameter sets were generated using the procedure described in Fig. S1 and the *Spartan* R package [20]. The steps are i) 10 unique baseline transmission parameter sets were drawn from previous model calibrations [10], ii) for each intervention scenario 100 new parameters were generated by sampling 100 unique parameter values from the distribution of the combined study data, iii) each set was mapped to one of the unique 10 transmission parameter sets to produce 100 new parameter sets. To reverse interventions where there was a single value, e.g., increased occupancy, each baseline transmission parameter set was replicated 10 times and then modified to include the single new parameter value in all sets.

These new parameters were used within the IBM to generate estimates of the impact of each intervention individually (in terms of nosocomial infections averted in inpatients and in HCW) as well as an overall estimate of the combined impact of all interventions that were implemented during the COVID-19 pandemic in England.

The model was simulated for 5100 timesteps covering a time period of 850 days from 03-March-2020 to 30-June-2022 (6 steps per simulated day). Individual-level patient and HCW data on infection status and location were recorded at each time step.

### Predictive modelling of the impact of combinations of interventions on infection rates

In a further analysis to explore the effect of intervention combinations, a list of all possible combinations of interventions described above was generated (255 total) and then explored. Unlike the previous analysis where vaccines and variants emerged during the pandemic and interventions were put in place at times specified by policy, here we restrict simulation to a 12-week Omicron-like period where the prevalence ranged from 2–4% (similar to January 2022), assuming no protection from previous infection or vaccination. This time period was selected to provide enough time to observe the effect of interventions while maintaining stable prevalence levels in line with community estimates. In this analysis all interventions were removed and then added back either singularly or as part of a package. Simulations were parameterised to reflect the hypothetical removal of all interventions except those included in the combination i.e., in a scenario where patient testing and LFD testing of HCWs were included, all other interventions are reversed as described in Table 1. Parameter files were generated as described above. The results of these simulations were then fed into a linear regression model to estimate the overall impact of each intervention. A second model was used to estimate the impact of combinations of the interventions that were significantly associated with a decrease in the number of infections in model 1.

$$\mathrm{Model}\;1:\;Lm\left(infections\sim{LFDs}_{ON}+Occupancy_{REDUCED}+\dots+HCW\_isolation_{ON}\right)$$

$$\mathrm{Model}\;2:\;Lm\left(infections\sim Intervention1_{ON}\ast Intervention2_{ON}\ast Intervention3_{ON}\right)$$

Results are presented as the number of susceptible inpatients and patient-facing HCWs becoming infected with SARS-CoV-2 under each intervention scenario across the simulation period. To scale up the single-hospital simulation estimate to a national level, the total number of infections prevented is divided by the total number of beds in the simulated hospital (a proxy for hospital size) and then multiplied by the total number of beds across NHS hospitals in England using national data [15]. This assumes that occupancy was similar between all NHS hospitals.

## Results

### Impact of interventions on nosocomial infections in patients: counterfactual modelling

In the baseline scenario where all interventions were in place, a maximum of 12,300 patients were infected in a single week and 240,000 over the simulation period (March 2020-July 2022, Fig. 1, Table 2, 3.5% of all susceptible admissions). Removing all interventions results in a worst-case scenario of up to 22,000 patients infected in a single week and 560,000 over the simulation period (6.25% %))of all susceptible admissions), a more than two times increase compared to the baseline. The most effective interventions were patient testing and universal masking by HCWs, and removing these interventions resulted in a total of 326,000 and 310,000 patient infections respectively, over the simulation period. Patient testing and universal masking by HCWs were most effective over the omicron wave (from December 2021), and testing was most effective over the first and second waves (March 2020 to May 2021). The least effective intervention overall was restricting visitation: removing this intervention did not result in an increase in infections; however, this intervention was only in place for a short time and therefore only has a limited opportunity for effectiveness (Fig. S2). In a scenario where visitation was restricted throughout the entire simulation period there were 44% fewer patient infections during the omicron wave (Fig. S3). Interventions were most impactful before July 2020 when the wild-type (WT)wave first hit England and between December 2021 and March 2022 when the omicron wave first started. This is demonstrated by the greatest difference between the simulations with no interventions (yellow lines) compared to the baseline (grey lines).

**Fig. 1:**
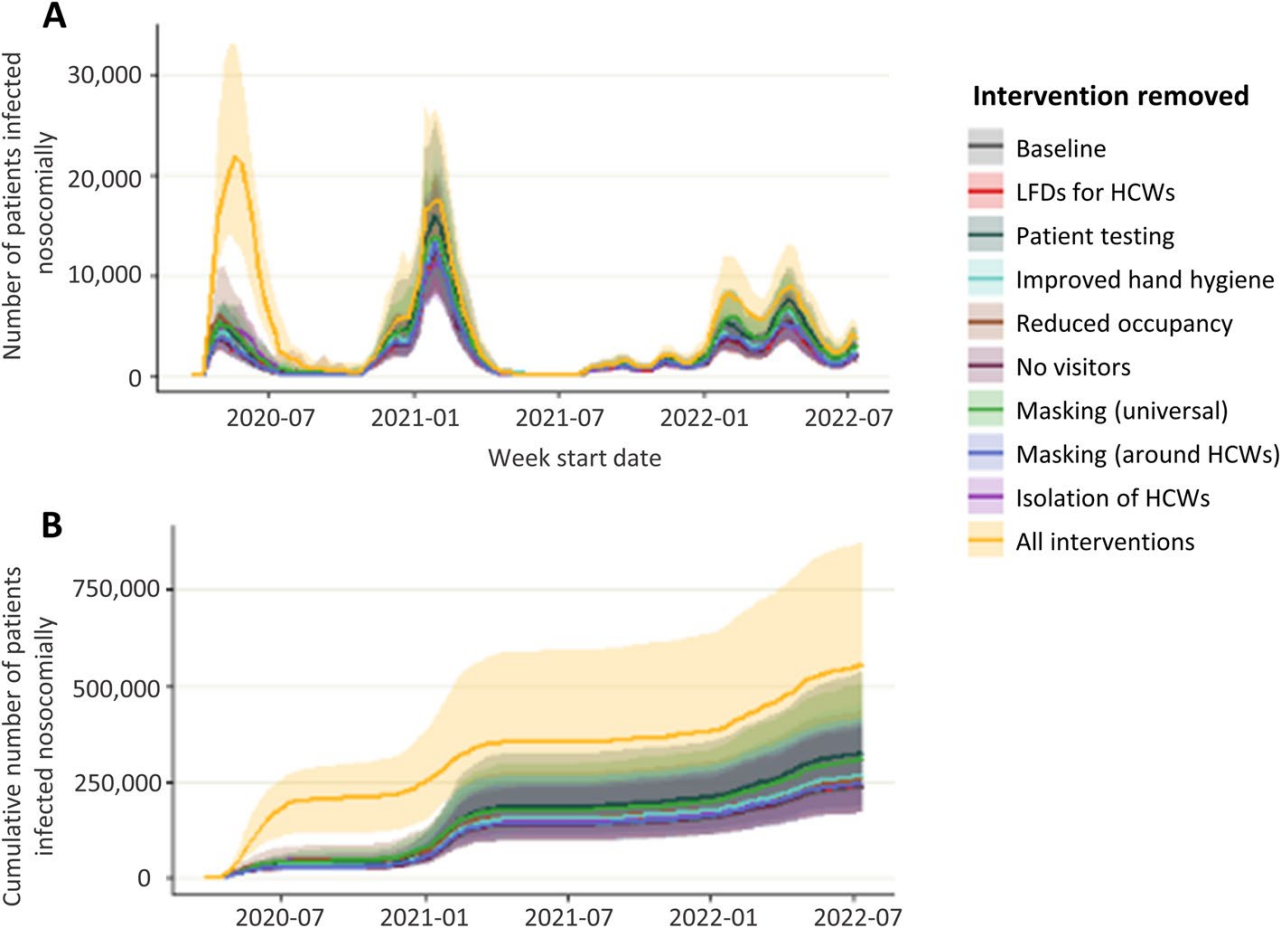
Number of nosocomial patient infections when single or all interventions are removed. Number of patients infected per week (**A**) and cumulative number of patient infections (**B**) when individual or combined interventions are lifted over the course of the pandemic

**Table 2:** Number of infections occurring in each of the simulated scenarios

	*Patients*				*HCWs*			
	*Total infections*		*Maximum number of infections in a week*		*Total infections*		*Maximum number of infections in a week*	
Scenario name	*Median*	*IQR*	*Median*	*IQR*	*Median*	*IQR*	*Median*	*IQR*
Baseline	240,000	(170,000–391,000)	12,250	(8,300–20,000)	595,000	(550,000–650,000)	17,500	(15,000–20,000)
HCW isolation	245,000	(186,000–395,000)	13,700	(9,000–20,900)	780,000	(750,000–850,000)	40,000	(35,000–50,000)
Testing	326,000	(225,000–535,000)	16,200	(12,000–26,900)	601,000	(561,000–663,000)	18,200	(15,900–21,000)
Occupancy	256,000	(189,000–436,000)	13,600	(10,000–19,400)	594,000	(557,000–645,000)	18,100	(16,300–20,000)
LDFs for HCWs	242,000	(169,000–403,000)	13,900	(9,400–21,200)	600,000	(561,000–654,000)	17,700	(15,900–20,000)
Hand-hygiene	268,000	(187,000–459,000)	14,500	(11,200–23,000)	595,000	(562,000–648,000)	17,800	(16,100–20,800)
Visitors	243,000	(169,000–390,000)	13,600	(9,300–20,500)	597,000	(558,000–646,000)	17,500	(15,800–19,700)
Masking (universal)	310,000	(230,000–500,000)	15,000	(11,100–22,100)	850,000	(770,000–960,000)	24,000	(19,000–30,500)
Masking (around HCWs)	246,000	(170,000–406,000)	14,600	(9,400–22,000)	750,000	(700,000–775,000)	18,000	(16,000–20,000)
All interventions	560,000	(420,000–875,000)	22,000	(13,000–33,000)	1,005,000	(956,000–1,068,000)	65,000	(50,000–75,000)

### Impact of interventions on HCW infections: counterfactual modelling

**Fig. 2:**
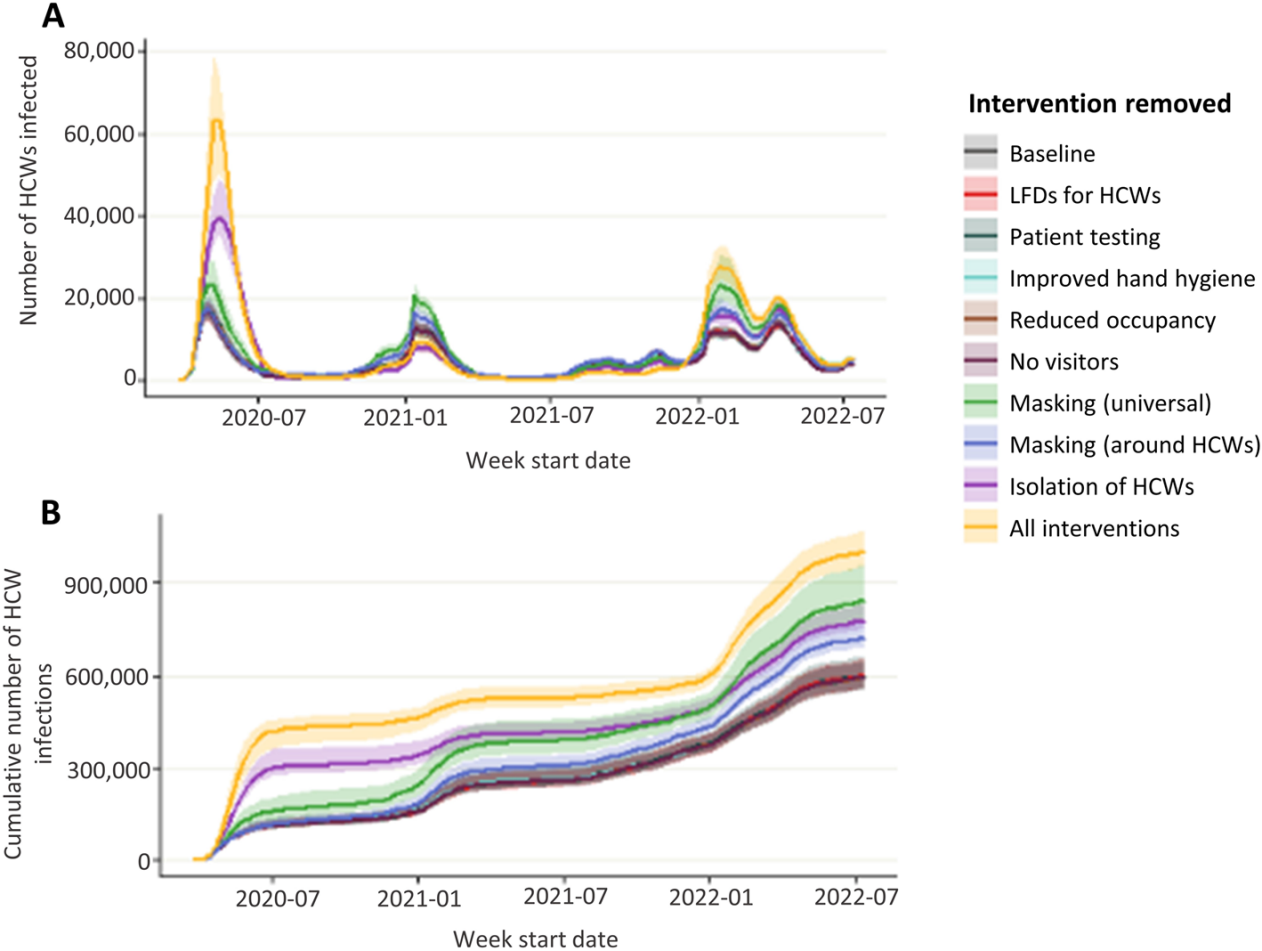
Number of HCW infections when single or all interventions are removed. Number of HCWs infected per week (**A**) and cumulative number of HCW infections (**B**) when individual or combined interventions are lifted over the course of the pandemic

**Fig. 3:**
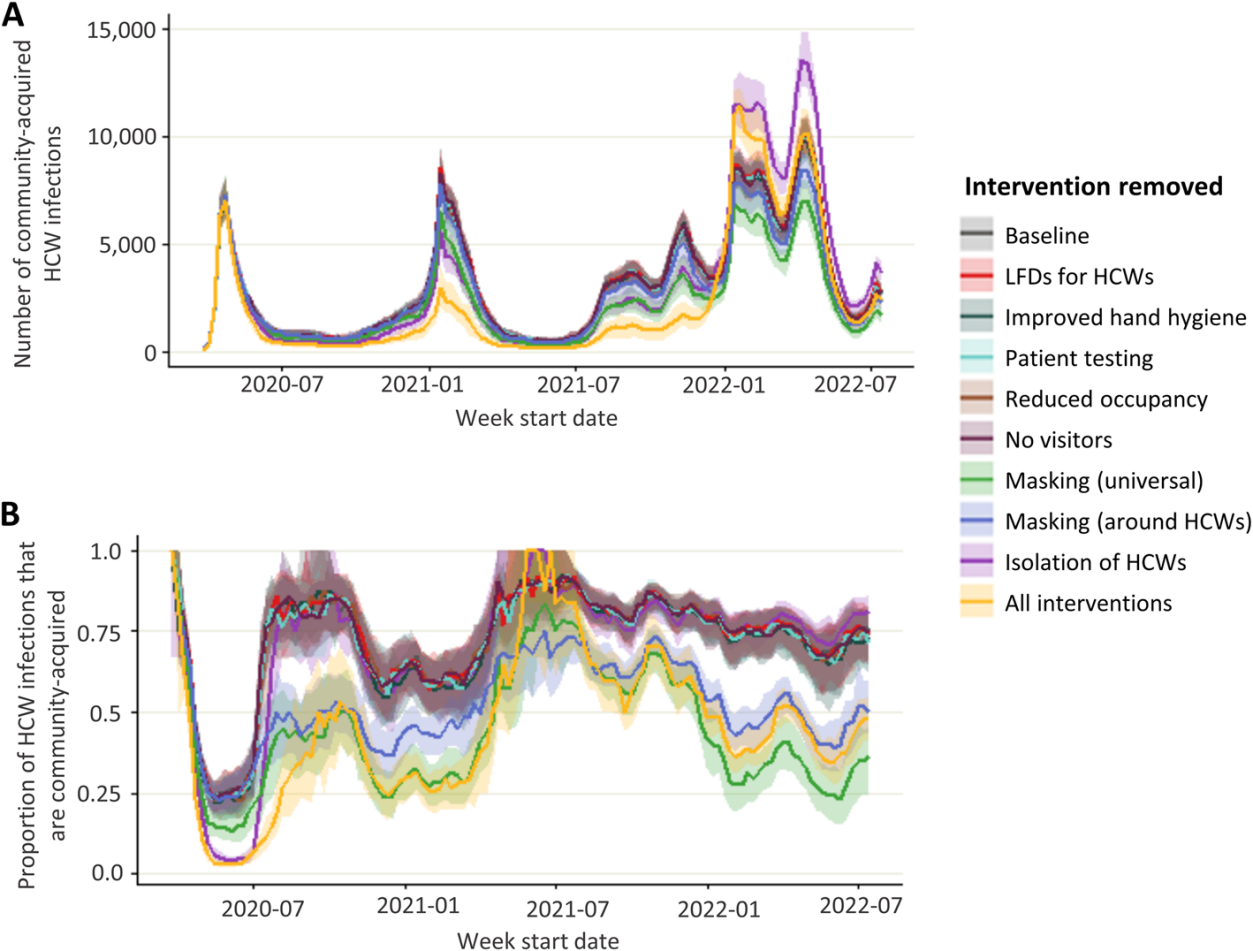
**A**) Community HCW infections **B**) proportion of community HCW infections

In the baseline scenario where all interventions were in place, a maximum of 17,500 (median, IQR in Table 2) HCWs were infected in a single week and there were 595,000 HCW infections over the simulation period (Fig. 2). When all interventions were removed simultaneously, a maximum of 65,000 HCWs were infected in a single week (10% of all staff) and there were 1 million (0.9 – 1.1 million) infection events in HCWs over the simulation period. The most effective interventions were isolation of symptomatic HCWs, masking universally, and masking around patients. When HCW isolation was removed, up to 40,000 HCWs were infected in a single week, and there were 780,000 infection events in HCWs. Removing masking universally resulted in a maximum of 24,000 HCWs infected in a single week and 850,000 infection events in HCWs. When masking was only in place around patients and not other HCWs a maximum of 18,000 HCW infections in a single week and there was a total of 750,000 HCW infection events. Masking was most effective during the first wave of the pandemic (March – August 2020), and HCW isolation was most effective both during the first wave and also over the omicron wave (from December 2021). When interventions are removed the main risk to HCWs becomes the hospital, with almost 75% of infections in HCWs being hospital acquired in 2022 (Fig. 3). Relaxing interventions switches the balance of infections from the community to the hospital.

### Impact of combined interventions on patient nosocomial infections: predictive modelling

**Fig. 4:**
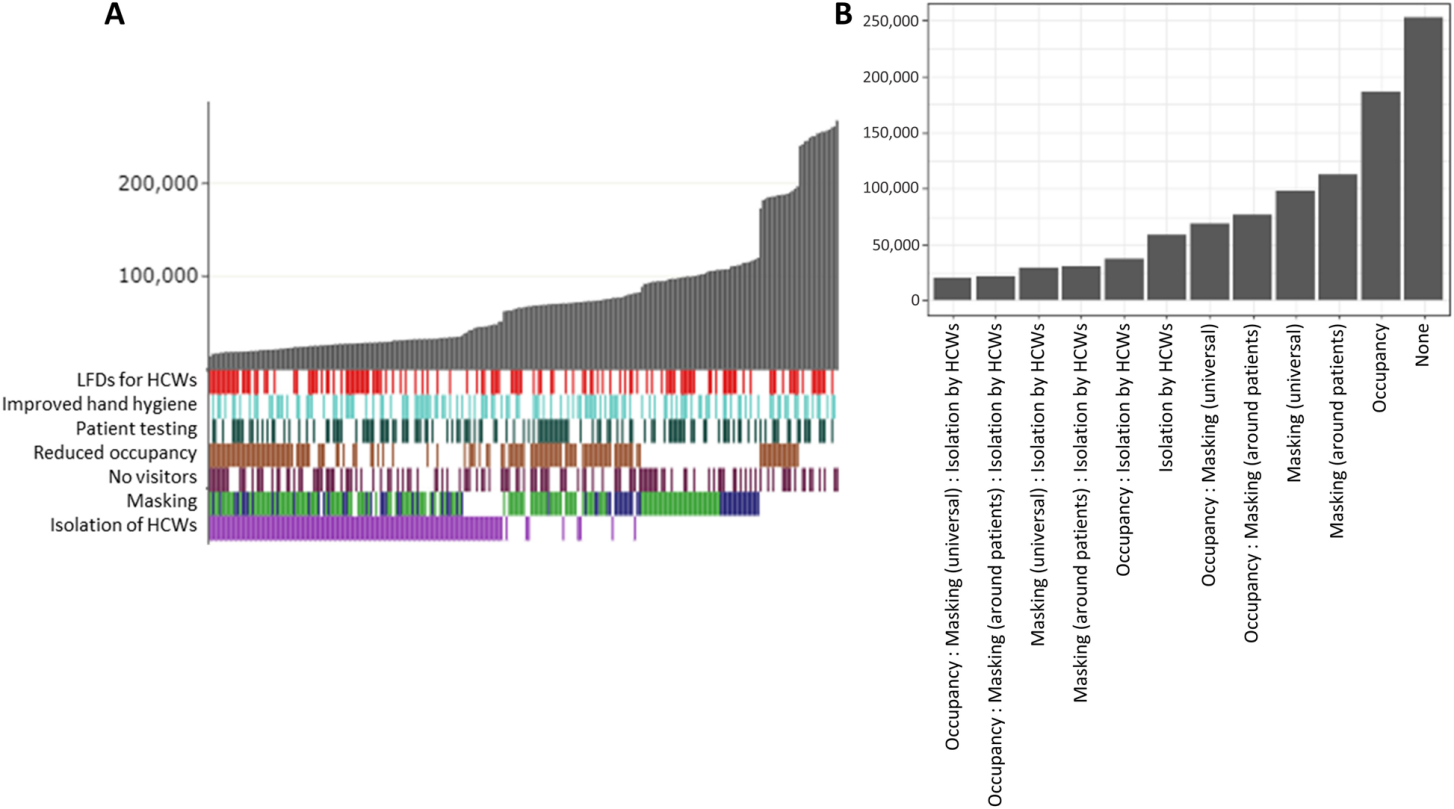
Modelled combined effects of interventions on patients. **A**) Simulated number of patients infected over a 12-week omicron-like period (grey bars) under package of interventions (coloured bars = interventions in package, white = intervention not in package) ordered by infection rate. For masking, green bars represent universal masking in package and blue represents only masking around HCWs. Each individual bar = single combination of interventions. **B**) Predicted number of patient infections over a 12-week omicron-like period under each combination of interventions from statistical model of simulated data

In a scenario with no protection from previous infection or vaccination where all interventions were removed, and then added back in either singularly or as part of a package, restricting visitation as a single intervention was the least effective option, with 270,000 patients nosocomially infected over a three-month period (Fig. 4A). The most effective single intervention was isolation of symptomatic HCWs preventing 81% of infections compared to restricting visitation alone (50,000 vs 270,000). When HCWs testing and isolation upon developing symptoms are in place, both universal masking and masking around patients significantly further reduce infections by up to 25% (36,000 vs 48,000) compared to restricting visitation alone, but there is no clear advantage to universal masking over masking only around patients for preventing nosocomial patient infections. However, when HCWs do not isolate then universal masking has a slight advantage over masking only around patients with an additional 11% reduction in infections (maximum of 107,000 infections when masks are worn universally vs 121,000 when worn only around patients).

A regression model on the simulated infection rates further validates these findings and identifies significant reductions in nosocomial patient infections when universal masking (β = -79,394.50, *p* < 0.001), masking around patients (β = -73,212.08, *p* < 0.001), isolation by HCWs (β = -89,522.22, *p* < 0.001) and, with smaller impact, reducing occupancy rates (β = -26,261.75, *p* < 0.001) (Table 3). A secondary analysis exploring the combined effect of the significant interventions (HCW isolation, reduced occupancy, and masking by HCWs either universally or around patients) demonstrates that isolation by HCWs has a larger effect on patient infections than a combination of masking and reducing occupancy and that combining all three interventions has the greatest effective (Table 3, Fig. 4B).

**Table 3:** Linear regression on number of patients infected when interventions were included in a package. Each intervention was represented as a binary variable (1 = included, 0 = not included) except masking that was a multi-level variable (2 = universal, 1 = around patients only, 0 = none)

Intervention	Beta	*P*-value
Intercept	195,060.57	0.000
LFDs	-4,156.57	0.212
Hand-hygiene	619.36	0.853
Patient testing and cohorting	-1,089.47	0.743
Occupancy	-26,261.75	0.000
Visitors	-2,017.43	0.544
Masks (universal)	-79,394.50	0.000
Masks (around patients)	-73,212.08	0.000
Isolation by HCWs	-89,522.22	0.000

### Impact of combined interventions on HCW infections: predictive modelling

**Fig. 5:**
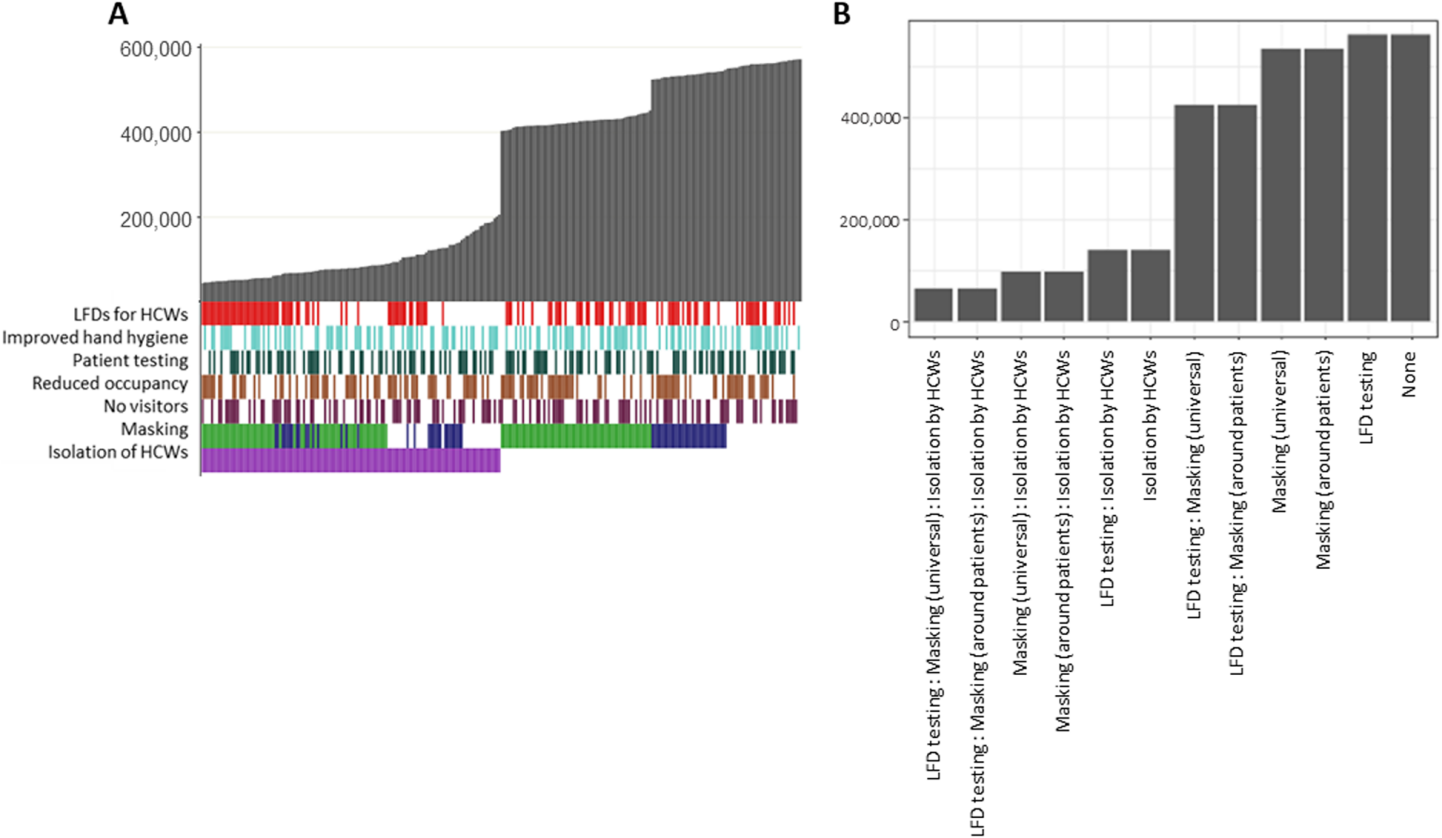
Modelled combined effects of interventions on HCWs**. A**) Simulated number of HCWs infected over a 12-week omicron-like period (grey bars) under package of interventions (coloured bars = interventions in package, white = intervention not in package) ordered by infection rate. For masking, green bars represent universal masking in package and blue represents only masking around HCWs. Each individual bar = single combination of interventions. **B**) Predicted number of HCW infections over a 12-week omicron-like period under each combination of interventions from statistical model of simulated data

For HCW infections, increased hand-hygiene alone was the least effective intervention resulting in 572,000 infections over a three-month period compared to a maximum of 206,000 infections under the most effective single intervention, isolation of symptomatic HCWs (Fig. 5A). Masking (both universal and around patients only) was also effective when HCWs do not isolate, with universal masking more effective than masking around patients (16% reduction in infections, maximum of 538,000 vs 451,000 HCWs infected). When HCWs do isolate, the difference in mask types is less pronounced and the impact appears to be related to other interventions e.g., LFD testing.

Statistical modelling of the simulated infection rates identifies significant reductions in transmission associated with lateral flow testing of HCWs (β = -22,464.66, *p* < 0.001, Table 4) universal masking (β = -106,902.84, *p* < 0.001), masking around patients (β = -34,552.93, *p* < 0.001), and isolation of symptomatic HCWs (β = -394,655.48, *p* < 0.001). There is also a small association with reduced occupancy (β = -8,2090.0, *p *= 0.007). A further analysis exploring the impact of the three most significant interventions combined demonstrates that isolation of symptomatic HCWs alone is more impactful than masking or LFD testing combined and that a combination of universal masking, LFD testing and isolation of symptomatic HCWs is the most effective way to prevent transmission (Table 4, Fig. 5B).

**Table 4:** Linear regression on number of HCWs infected when interventions were included in a package. Each intervention was represented as a binary variable (1 = included, 0 = not included) except masking that was a multi-level variable (2 = universal, 1 = around patients only, 0 = none)

Intervention	Beta	*P*-value
(Intercept)	563,606.48	0.000
LFDs	-22,464.66	0.000
Hand-hygiene	1,154.87	0.703
Patient testing and cohorting	-1,244.54	0.681
Occupancy	-8,209.00	0.007
Visitors	863.66	0.776
Masks (universal)	-106,902.84	0.000
Masks (around patients)	-34,552.93	0.000
Isolation by HCWs	-394,655.48	0.000

## Discussion

We evaluated the counterfactual impact of removing any or all of eight interventions that were implemented in NHS England hospitals over the COVID-19 pandemic on rates of nosocomial transmission to patients and HCWs in England. While highly uncertain, given the dependency on variable evidence on the effectiveness of interventions, the model results suggest that interventions in place over the COVID-19 pandemic in England prevented over 50% of potential nosocomial infections in patients and HCWs. Testing and cohorting of patients and isolation of HCWs were the most important interventions for reducing transmission to patients and HCWs preventing up to 34% (30–40%) of infections. We also identified a significant role for masking, with universal masking being more impactful than masking around patients alone (40% (30–52%) vs 17% (14–20%) reduction). Results suggest that restricting visitation could be impactful when community prevalence rates are high. Interventions were most impactful when protection from vaccines or previous infection was low at the start of the wild-type and omicron waves and had little impact in times of high immunity.

To the best of our knowledge this is the first study to attempt to quantify the impact of IPC measures over the COVID-19 pandemic in a hospital setting. A literature search conducted to parameterise the model in this study highlighted a paucity of evidence around the effectiveness of interventions such as improving hand-hygiene, as demonstrated by other studies [21], and a wide degree of uncertainty in the effectiveness of others such as masking on reducing nosocomial transmission of respiratory viruses. Further there is a lack of evidence around compliance with any interventions that were implemented. While the model used in this study includes the effect of vaccines, as administered during this period, on both patients and HCWs, we do not evaluate the impact of alternative vaccine availability or uptake. The modelling approach used in this study has previously been used to estimate the counterfactual HCW infections assuming absence of vaccines in the second wave of the pandemic and found prioritising HCWs for vaccinations was extremely important for reducing infection rates in HCWs [9].

While the baseline results are calibrated to high-quality national datasets, the scarcity of reliable evidence on the effectiveness of individual interventions (required for simulating a scenario where they are reversed) is a key challenge. While the model has been parameterised to best reflect the available data, uncertainty remains around the contribution of nosocomial and community sources of SARS-CoV-2 infection. As COVID-19 becomes an endemic disease and pressures on health systems from other seasonal respiratory pathogens increase, there is a critical need for evidence on the effectiveness of such interventions on reducing nosocomial spread in order to design efficient and effective infection prevention and control strategies.

In this study we assumed full compliance with interventions such as testing and isolating (although the compliance in studies used to parameterise masking and hand-hygiene was not reported), and the impact of reversing an intervention was applied to every interaction at every timestep that the intervention was in place. If compliance was poor or changed throughout the study period, then the modelled impact of an intervention would fall. In the absence of data on policy implementation across trusts over time, we assumed uniformity between trusts in changing behaviours according to policy guidelines. When off shift, the infection risk for HCWs was uniform, and we did not account for individual-level differences in risk of infection in the community. Further, we assumed that the effect of interventions was the same regardless of the strain on SARS-CoV-2 that was circulating at the time. We also made the assumption that it is appropriate to scale up the average results from our simulations to a national level using only numbers of beds therefore implying that all trusts were similar in terms of occupancy and admissions rates throughout the pandemic. If this was not the case, the results would need to be viewed at an individual hospital level, and although we expect the general trends to hold, the magnitude of interventions' effects may change. Similarly, for HCWs we scaled the results to a national level using total patient-facing HCW counts alone and assumed that the infection risk for staff off-shift was uniform which may not be true in reality and we could be under or overestimating the impact of interventions such as masking if staff were more or less risk-averse than the general population when off-shift. Due to policies being implemented universally and in quick succession at the start of the pandemic, there is limited opportunity to analyse their efficacy from a data-driven perspective; however, a small number of studies exist that validate the results of this modelling study. A single-site study showed that between the first and second waves the proportion of nosocomial infections in HCWs attributable to HCW-to-HCW transmission fell from 55.3% in wave 1 (01-March-2020 to 25-July-2020) to 37.4% in wave 2 (30-Nov-2020 to 24-Jan-2021) despite the proportion of community-acquired infections remaining at 50% [2]. This suggests that the introduction of masks in communal spaces in June 2020 potentially played a role in reducing HCW-to-HCW transmission by as much as 32%. This is consistent with our estimate that over wave 1 an additional 50% (27%-57%) of HCWs would have been infected had universal masking not being introduced. Another study examined the impact of removing HCW masking on patient infections and did not see a significant increase in infections when masks were removed during the first 10 months of omicron when immunity from previous infection and/or vaccination was high [22]. This agrees with simulation results where an increase in patient infections in the absence of masking was observed in the wild-type and early omicron waves, but there is little difference when vaccination and immunity from previous infection has increased protection. We did not find a notable increase in nosocomial patient infections following the withdrawal of asymptomatic testing of patients and HCWs or of masking late in 2022.

This analysis identified testing and isolation of symptomatic HCWs and masking by HCWs around both patients and other HCWs as the most important interventions for reducing infections in the patient and HCW populations. There is evidence from early in the pandemic that HCW-to-HCW transmission commonly occurred and the risk of HCW-to-patient transmission is likely small [2–4, 10]. However, there remains a potential for a larger effect of a small number of HCW-to-patient transmissions seeding larger outbreaks on patient wards. This cannot be ignored, and likely contributes to the increased impact of interventions that minimise HCW-to-patient transmission. However, there remains a. This impact. A strength of the IBM is that it captures these ‘knock-on’ benefits associated with prevention of transmission chains both within and across patient and HCW populations; such combined/bundled effects are sometimes referred to as the ‘Swiss Cheese’ infection prevention model [23, 24]. This cumulative effect of reducing transmissions is apparent when looking at the impact of removing all interventions in combination, which results in a higher number of nosocomial infections than the sum of the individual interventions. Model findings suggest that collectively the interventions introduced over the SARS-CoV-2 pandemic in England averted 400,000 (240,000 – 500,000) infections in inpatients and 410,000 (370,000 – 450,000) HCW infections.

Masking was found to be a highly effective intervention, but due to a lack of studies exploring the impact of different mask types we consider the overall impact of any masking in this study and do not distinguish respirators from fluid resistant surgical masks (FRSMs). A previous modelling study suggested that respirators may be advantageous for preventing infections in HCWs; however, the authors explicitly state that their study provides evidence that more work should be done to determine the true effectiveness of respirators over FRSMs [25], and no further convincing evidence has yet emerged. Analysis of intervention reversal only considered those described; additional measures such a double gloving or sessional gown use are not included in this work. The impact of interventions such as increasing bed spacing and improving ventilation were not modelled as their impact could not be included in the existing modelling framework upon which this study was conducted. While we have attempted to estimate the impact of results on infection rates, the adverse impact of interventions such as restricting visitation or mask wearing on patient care and staff wellbeing have been well documented and cannot be ignored [26–36]. This extensive literature should be considered alongside the results of modelling work when making future policy decisions.

## Conclusion

This study combines a transmission model with published parameter estimates on the impact of individual measures to evaluate the contribution that the collection of interventions and hospital changes in place over the COVID-19 pandemic in England made to the reduction of nosocomial transmission, quantifying both individual intervention and collective impact. A strength of the modelling approach used here is the ability of the model to capture cumulative effects of interventions through reducing the seeding of new infection clusters. These results highlight the importance of maintaining high levels of compliance to infection prevention and control measures in hospitals and have important implications as hospitals prepare for a surge in demand due to emerging winter pressures and COVID-19.

### Supplementary Information

**Supplementary file 1. **

**Supplementary file 2. **

**Supplementary file 3. **

## Data Availability

All UKHSA-held data (from SUS and SGSS as detailed in Supplementary File 1) were collected within statutory approvals granted to UKHSA for infectious disease surveillance and control. Information was held securely and in accordance with the Data Protection Act 2018 and Caldicott guidelines. The data that support the findings of this study are available from NHS Digital but restrictions apply to the availability of these data, which were used under license for the current study, and so are not publicly available. The datasets used and/or analysed during the current study are however available from the corresponding author on reasonable request and with permission of NHS Digital.
