# Supplementary material for: Impact of interventions to reduce nosocomial transmission of SARS-CoV-2 in English NHS Trusts: a computational modelling study
Source: BMC Infect Dis. 2024 May 7;24:475. doi: 10.1186/s12879-024-09330-z (PMC11075183; doi:10.1186/s12879-024-09330-z)
Supplement: Supplementary file 1 [file 12879_2024_9330_MOESM1_ESM.docx]

**Efficacy of interventions to reduce nosocomial transmission of SARS-CoV-2 in English NHS Trusts: A computational modelling study**

**Mathematical model methodology**

**High-level model description**

8000 HCWs work 12 hour shifts (~50% on shift at any one time)

30% HCWs only work on a single ward

Other HCWs are transient and visit 18 patients a shift^1^

In the absence of testing, symptomatic staff have a 60% chance of self-isolating following the development of symptoms^2^*1. Cohen (2012). 2. Kluytmans-van den Bergh (2020)*

**
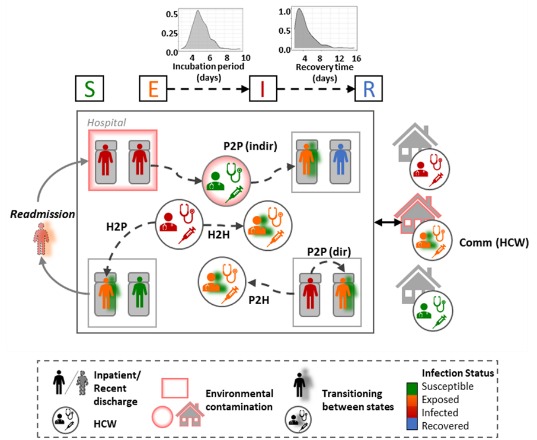

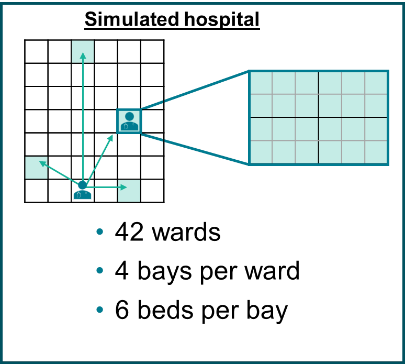
**

**Patients can become infected via transmission from:**

***P2P (dir) - Patients in the same bay
P2P (indir) - Patients on the same ward but not necessarily the same bay
H2P – HCWs during treatment***.

**HCWs can be infected via transmission from:
*P2H* - Patients they are treating
P*2H (dir)*– HCWs that have visited the same ward in the last time step (4hrs) *H2H (indir)*– HCWs anywhere in the hospital
*Comm (HCW)* – The community while off shift**.

**An individual-based model of the spread of COVID-19 in English hospitals**

# **ODD Protocol**

We have developed an individual-based model (IBM) of the transmission of COVID-19 in an English hospital and use the Overview, Design concepts, and Details (ODD) protocol^1^ to provide a complete and rigorous model description of the model used for the simulations described in the Mathematical Model” section of the main document methods.

## Purpose

The purpose of the model is to represent the acquisition and transmission of SARS-CoV-2 by patient-facing healthcare workers (HCWs) and patients in a typical English hospital and answer questions around the source of infection and the effectiveness of interventions.

## Entities, state variables and scales

The model contains two environment classes, namely the Community and the Hospital. The Community class tracks the change in community prevalence per day and determines the number of community-acquired symptomatic and exposed cases to admit to hospital. The Hospital class stores the agent lists and handles the creation of newly admitted agents as well as the removal of patients that are to be discharged. There are two types of agents in the model, Patients, and HCWs, both of which inherit basic variables from the abstract People class.

Table 1: States and variables of model agents

| Variable | Definition |
| --- | --- |
| **Community** |  |
| prevalence | Proportion of community infected on a single day. Calculated from Cambridge RTM (<https://www.mrc-bsu.cam.ac.uk/tackling-covid-19/nowcasting-and-forecasting-of-covid-19/>) and regional population estimates from NOMIS (<https://www.nomisweb.co.uk/home/release_group.asp?g=8>).  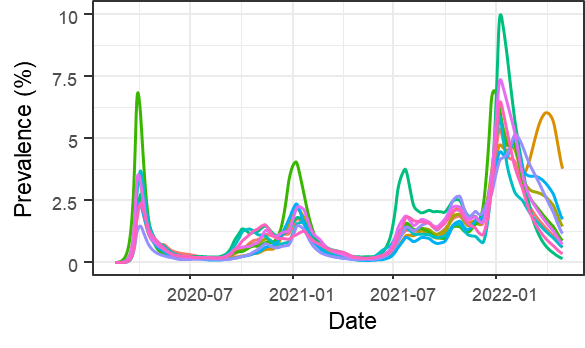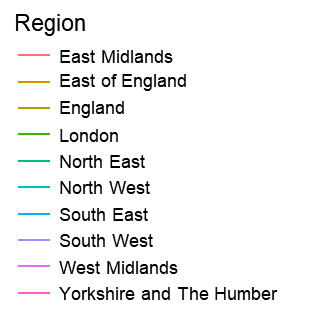 |
| infCases | Number of people currently infected. It is the product of population size and prevalence |
| expCases | Number of people exposed in community  *infCases** 1/(1-asymptomaticProb) |
| region | Region to be simulated from:  "North West", "North East and Yorkshire", "South West", "South East", "London", " Midlands", "East of England" |
| prop_{alpha, delta, omicron} _comm/hosp | Proportion of cases in the community or hospital admissions that are with each variant. |
| ld_{alpha, delta omicron} | Logistic distributions for proportion of community-acquired hospital cases that are of each variant per time step. |
| ld_vacc_age_group{1,2,3} | Logistic distributions for vaccination probability for each dose in new admissions. Age groups are: 16-49, 50-54,55-59, 60-64, 65-69,90-74, 75-79, 80+. |
| **Hospital** | |
| hospital | Array list containing *Parameters.wards* ‘wards’ that are also array lists, each of which contains *Parameters.shared_bays* ‘bays’ (also array lists) that contain *Parameters.bedsPerBay* ‘beds’, where each position in the beds array list either contains a single patient agent or is set to null. |
| red_wards | Array list of the position of those wards that contain known or suspected COVID-19 patients in the hospital ward array list |
| green_wards | Array list of the position of those wards that contain no known or suspected COVID-19 patients in the hospital ward array list |
| red_to_green_wards | Array list of the position of those wards to move from red to green at the end of the time step |
| green_to_red_wards | Array list of the position of those wards to move from green to red at the end of the time step |
| hcws_on_wards | Array list containing a separate array list for each ward that records which HCWs have visited that ward in a time step |
| infectedPatientsByBay | Map of the number of patient agents in each bay that are infected |
| **People** |  |
| id | Unique identifier for agents |
| Infection_Status | Enum for infection states. One of:  NONE (never infected),  EXPOSED (infected but not yet detectable or infectious. After a pre‑defined incubation period these individuals transition to one of the infectious states),  INFECTED_P (pre‑symptomatically infected, PCR-detectable and infectious but not yet symptomatic. These individuals become symptomatically infected after a pre‑defined onset period has passed),  INFECTED_A (asymptomatically infected, PCR-detectable and infectious),  INFECTED_S (symptomatically infected, PCR-detectable and infectious),  RECOVERED (recovered/immune, non-infectious, assumed to be a permanent state). |
| Infection_status | Current infection status |
| recoveryStep | Timestep to recover from infection (if no infection then 1000000000) |
| incubationPeriod | Timestep to transition from exposed to pre-symptomatic (if no infection then 1000000000) |
| onsetPeriod | Timestep from pre‑symptomatic to symptomatic (if no infection then 1000000000) |
| Type | Enum for type of person agent (Patient/HCW) |
| type | Type of this agent |
| Gender | Enum for gender. Male/Female |
| gender | Gender of person agent |
| communityInfection | Boolean for whether agents have a community acquired infection or not |
| timeInSim | Length of time agent has been in simulation |
| infected_by | Type and ID of agent that caused the infection (or Comm if infected in the community). |
| infection_type | Direct or indirect |
| vaccinated1 | Boolean for whether person ever received a first dose of vaccine |
| vaccinated2 | Boolean for whether person ever received a second dose of vaccine |
| vaccinated3 | Boolean for whether person ever received a third of vaccine |
| infected | Boolean for whether person was ever infected |
| vaccinationDay1 | Day that individual will become (HCWs) or became (patients) effectively vaccinated following a first dose of a vaccine |
| vaccinationDay2 | Day that individual will become (HCWs) or became (patients) effectively vaccinated following a second dose of a vaccine |
| vaccinationDay3 | Day that individual will become (HCWs) or became (patients) effectively vaccinated following a third dose of a vaccine |
| asymp | Boolean for whether an infection is asymptomatic or not |
| ct | Current CT value |
| ct_max | Ct-value at peak, drawn from truncated normal distribution |
| ct_start | Time that ct-value is first below ct_max |
| ct_max_step | Time step when ct-value will reach peak drawn from gamma distribution |
| ct_end_step | Time step when ct-value will return to pre-infection level |
| StrainCarried | Enum for possible strains to be carried (ALPHA, DELTA, OMICRON, OTHER} |
| strain_carried | Strain carried by infected individual |
| **Patients** |  |
| Ward | Enum for ward type. One of: NEW_ADMISSION, GENERAL, SUSPECTED, TEST_NEG, TEST_POS, DISCH |
| dischargeTimeSteps | The time in steps that a patient will be discharged |
| knownInfected | Boolean for identified infection |
| readmission | Boolean for whether patient has been readmitted or not |
| timeSinceDisch | Number of timesteps that the patient has been discharged for |
| carehome | Boolean for whether patient is from a care home or not |
| followOnDisch | Boolean for whether patient should be followed up on discharge or not. |
| tested | Flag for whether patient was tested again after 5-7 days (to ensure they only get tested once in this interval) |
| visitorsPerStep | Variable indicating the probability of having a visitor from the community during each time step and subsequently being infected |
| **HCWs** |  |
| onShift | Boolean for whether HCW is currently on shift or at home |
| absentThroughSickness | Boolean for whether HCW is currently isolating |
| stepToReturn | Timestep to end self-isolation and return to work |
| onRedWard | Boolean indicating whether a HCW is working on a red ward |
| FFP3 | Boolean variable for FFP3 wearing (over FRSM) on green wards |
| shiftStartStep | Timestep that shift began or will begin if currently off shift |
| shiftEndStep | Timestep that shift ended or will end if currently on shift |
| tester | Boolean variable for whether HCW undergoes asymptomatic testing |

## Process overview and scheduling

The model runs with a 4-hour time step, i.e. six time steps per day. Currently all agents are on the same time step schedule and are handled by the agent scheduler built into the Multi-Agent Simulator of Neighbourhoods toolkit (MASON)^2^ that randomly shuffles agents so that there is no order of which class/agent is stepped first, so the order of the processes active at every time step described here is arbitrary.

***Community*:** When a community object is instantiated, it randomly selects a region and reads in the admissions data for that region from the file “region_name.csv”. Every time the community object is stepped it performs three basic tasks. It updates the prevalence variable in line with the admissions data that has been read in, calculated the number of infected cases in the community, and calculated the number of exposed cases in the community. The parameters for the logistic curves defining the proportion of hospital cases that are with the specific variants are different between regions. This distribution and the vaccination probability over time by five-year age bands are generated when the community object is instantiated.

***Hospital*:** At the very first time step the function *setupHospital* that reads in the number of HCWs and beds from the parameter file and creates all the HCWs and patient agents required, each with *infection_status* = NONE, and randomly assigns each HCW’s *onShift* variable. At each subsequent timestep the number of infected, susceptible, and exposed to admit from the community in this 24-hour period is calculated. The number of infected patients to admit is taken from the admissions file in the community object, and the *communityInfection* variable is set to true, then for a random number of the remaining empty beds patients are admitted as either susceptible or exposed. The exposure probability is proportional to the number of infected patients being admitted at that time (*Patients.expOnAdmissionProb* in the parameter file). Next the hospital’s ward classifications are updated: making any red wards that have no known or suspected COVID-19 patients green and any wards with known or suspected COVID-19 patients red. Finally the list of HCWs that visit the ward in that time step is reset ready for the next iteration of the hospital. On admission patients are assigned a length of stay (LOS) from a Weibull distribution that is a function of their age, gender, and infection status. The hospital is divided into *wards* “wards” with *shared_bays* “bays” in each ward and *bedsPerBay* beds in each bay (default *bedsPerBay* = 6). There are two locations for HCWs in a hospital instance, either in the hospital (can interact with all other HCWs in the hospital) or out of the hospital (implicitly interact with the community), and HCWs that are in the hospital interact with patients by randomly selecting beds within bays within wards to visit, during which time they interact with only the patient in that bed and no other patients or HCWs. Other spatial aspects of a hospital (wards, corridors, offices etc) are not explicitly represented. A subset of HCWs are defined to be *wardBased*, those HCWs only select a bed/bay to visit since their activities are restricted to a single ward defined on admission.

***People*:** At each timestep every person agent which has *infection_status* = NONE calculates the probability of becoming exposed based on the number of infected patients they are exposed to on each ward, the number of infected HCWs they are exposed to in the hospital (randomly sampled from the number of infected HCWs and non-infected HCWs), and the transmission rates bP2P, bP2H, bH2H and bH2H from the parameter file. The transmission probabilities are assumed to be the absolute probability of transmission across all possible modes (airborne, aerosol, fomite) from each specified source. People that have *infection_status* = EXPOSED transition to the next infectious state according to their Ct values that are calculated using the Kissler model [1], the values from the Ct-value model are then drawn from the distributions described by [1]. People are assigned to a symptomatic or asymptomatic pathway on exposure, with a Ct value of 30 used for the threshold for infectiousness in either case [2] and symptom onset time coinciding with the maximum Ct value as described by Quilty et al [3]and parameterised using data from the literature [1,4,5](See Submodels for more detail). When the Ct value is less than *ct_max* People are assigned *infected_status* = INFECTED_P and when the Ct-value peaks they become INFECTED_S. When the recovery timestep is exceeded, people have *infection_status* RECOVERED, and are immune to future infections. If the person agent is a Patient then when they become infected a new LOS is generated. Currently there is no rule stating that this should be greater than the original LOS. Note that the recovery timestep can be greater than the LOS.

***Patients:*** As well as undergoing the behaviours that apply to all People agents in the hospital, patients can be tested on admission and are assigned to either single rooms or multiple bed bays, selected following the flow diagram in Figure 1. They are also discharged and those that have an *infection_status* of EXPOSED or INFECTED_P on admission are stepped for a further 14 days. During this time, the same Patient agent may be readmitted to hospital. On admission patients may have been vaccinated with one or two doses of a vaccine, with the vaccination probability being drawn from the dose-specific logistic distribution for their age group from the Community class. We took vaccination data from National Immunisation Management System (NIMS), with population denominators taken from Office for National Statistics (ONS) population predictions for 2020^3^, stratified for age-band included in the Joint Committee on Vaccination and Immunisation (JCVI) priority list^4^. It is assumed that COVID-19 admissions are vaccinated according to the average rates in the population for their age group. Vaccine efficacy wanes at a rate of *vaccine_waning_per_step* until it reaches 10%, estimated from [6] (See *Submodels* for more information). Infection with WT alpha, or delta variants conveys complete protection against other non-omicron variants but only partial protection against infection with omicron strains. Patients that share a bay with an infected patient can be infected directly with transmission probability *bP2P* and all patients in the ward can become infected indirectly by any other infected patient based on the number of infected patients in the hospital with probability *P2P_hosp*. Patients can also be directly or indirectly infected by any infected HCW that is on shift with probability *bH2P,* or indirectly by a patient where there is shared HCW contacts again with probability *bP2P_hosp*. We have calibrated these probabilities to reproduce observed data on the proportion of inpatients infected at an individual trust level. Patients can also be infected by visitors in periods when visitation is allowed. The probability of being infected by a visitor is 0.021 per timestep and the transmission probability was estimated based on the length of the visitation [7] (elicited from experts).

***HCW*:** In addition to the behaviours exhibited by all People agents, HCWs iterate between on and off shift, where they are infected by either other people in the hospital (if *onShift* = true) or with a probability of 0.13*beta*community prevalence per day if *onShift* = false . This is derived from data stating that each case has on average seven contacts. If 1.4 million/66.4 million people work for the NHS^5^ that means that ~2% of people in England are classed as HCWs in this setting, therefore the probability of one of these seven contacts being a HCW is 1 – (probability none of them are) = 1- 0.98^7^ = 0.136. For each contact the transmission rate is approximately 0.4 thus the probability of a HCW picking up an infection in the community is 0.136*0.4*community incidence rate (prevalence/4.6 days) where 0.4 is the transmission rate in the community^6^. HCW agents can also self-isolate while their *infection_status* is INFECTED_S, they can also be periodically tested with lateral flow device (LFD) tests and those with positive results are forced to isolate, with two days isolation following a false positive or ten days isolation following a true positive. HCWs that have been isolating following a true positive are automatically classed as RECOVERED when returning to work. The time that a HCW will become vaccinated (for first and second doses) is assigned on instantiation and is selected from dose-specific logistic distribution, we calculated the distribution function parameters from SIREN study participants linked with NIMS.

va

| 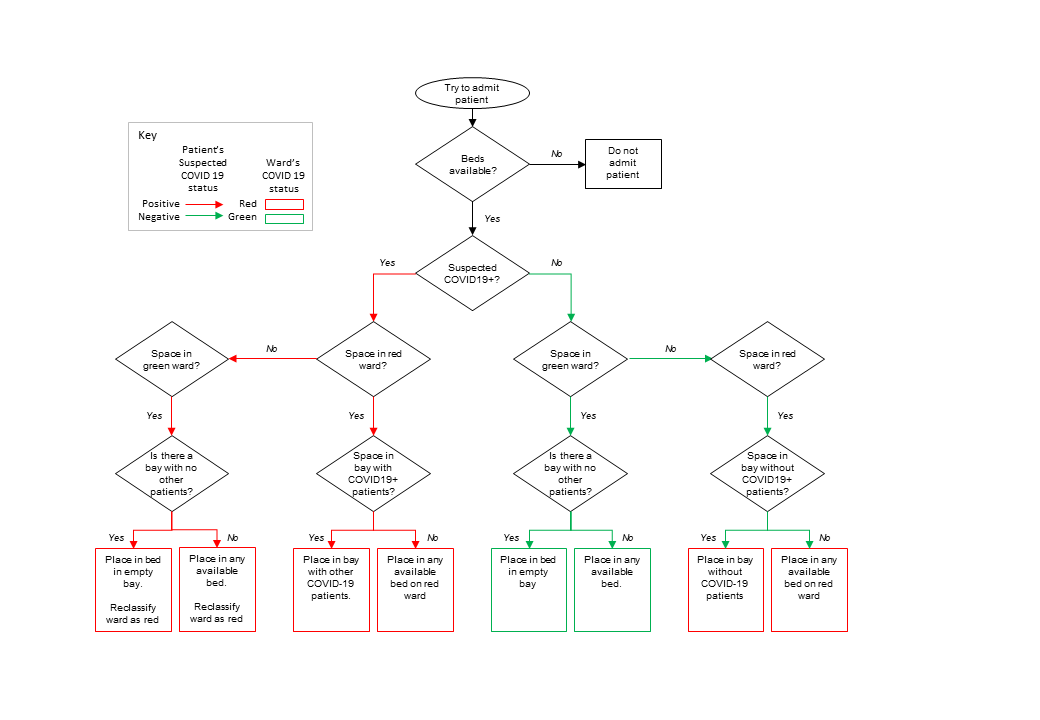 |  |
| --- | --- |

Figure 1: Schematic of assignment of patients to beds

## Design Concepts

The model is designed with the structure demonstrated in Figure 2A.

The emergent properties of this model are number of infected patients and HCWs over time and the sources of infection. Parameter uncertainty is introduced by using probabilities for infection rates and the inclusion of LOS, incubation, onset, recovery, and vaccination variables drawn from probability distributions.

We parameterised this model for a typical English hospital with 1000 beds and 8000 HCWs to reflect the average bed size to staff ratio in NHS England (NHSE) Trusts. We estimated death rates and length of stay distributions using the R library *fitdistrplus*^7^ and data from the Secondary Uses Service (SUS) COVID-19 linked to laboratory data of positive tests from Public Health England’s Second Generation Surveillance System (SGSS) (methodology as in Bhattacharya et al.^8^). The death probability was estimated by fitting a polynomial to the average probability of dying in hospital per year of age multiplied by the discharge probability from the SUS dataset, sampling the age distribution from the SUS dataset, and taking the mean. For susceptible individuals the death probability was calculated from the literature^9^ and for infected individuals, the combined SUS/SGSS data were used. Data gathered from SUS comprised all completed hospital spells in NHSE Trusts arising from admissions from 10 –March-2020 through to 30-June-2022 inclusive. We obtained age (in years) and sex of the patient was obtained, and calculated length of stay to the nearest day using recorded admission and discharge dates. We fitted length of stay distributions for positive and negative admissions using a Weibull distribution and adjusting for age and sex. Suitable values for transmission parameters between patients and HCWs were selected to quantitively and qualitatively reproduce data from individual Trusts in different regions of England, with differential case-loads. HCW infection data was calibrated using a meta-analysis of studies on HCW infection rates^10^ combined with results from the SIREN study.

| **A**  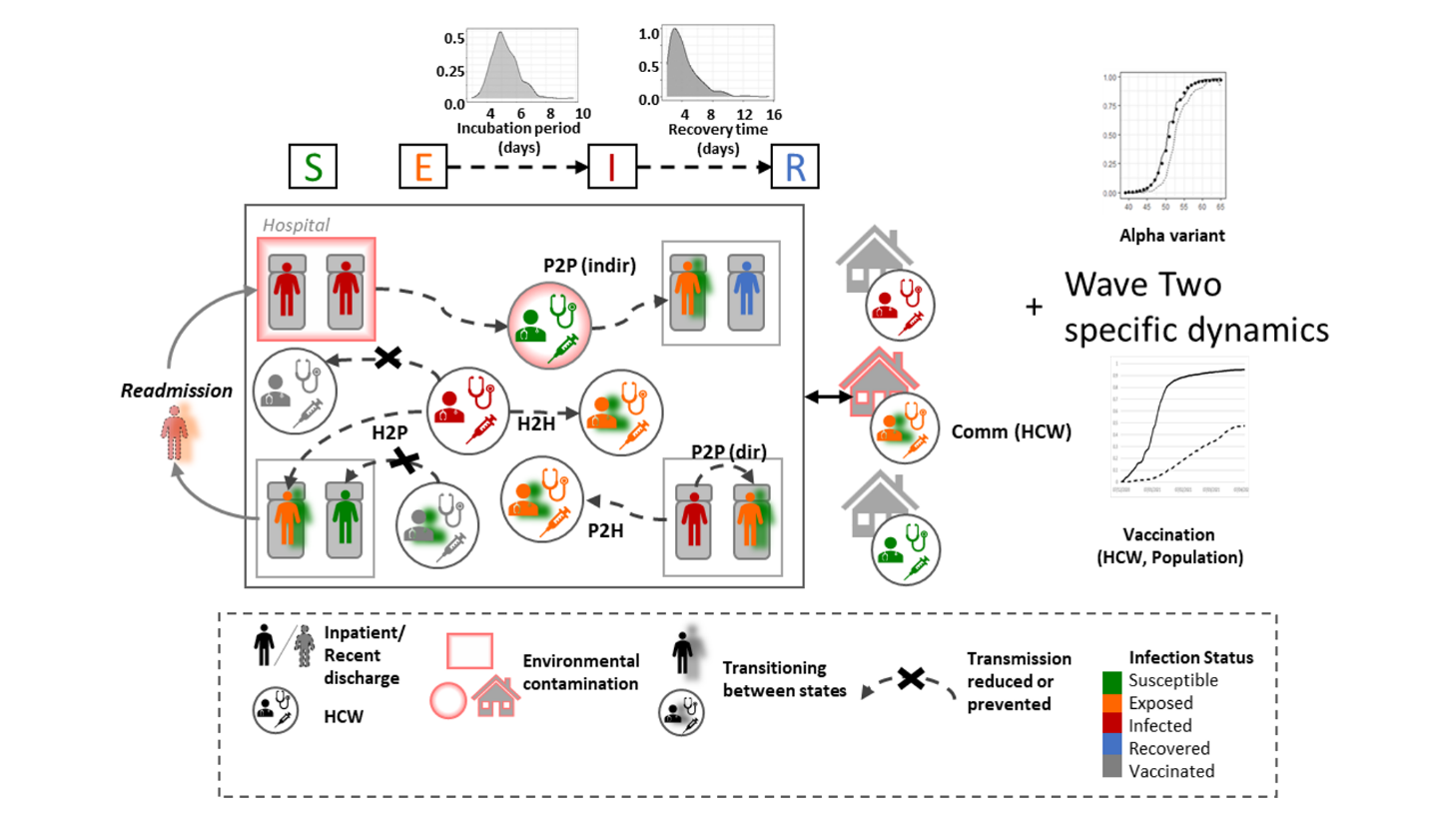 | **B** | 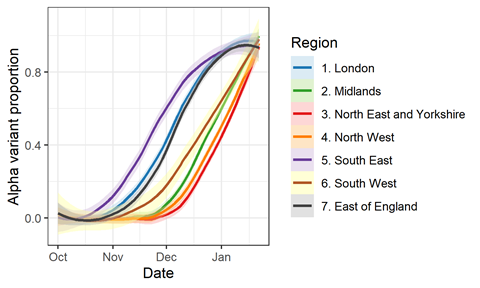 |
| --- | --- | --- |
|  | **C** | 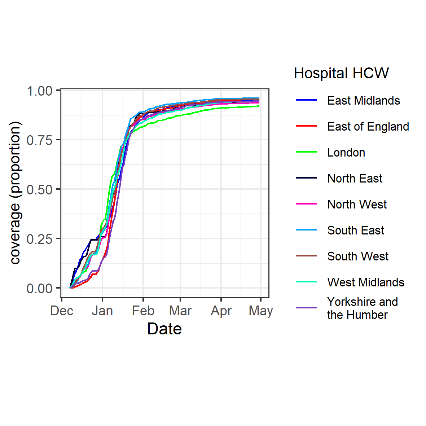 |
|  | **D** | 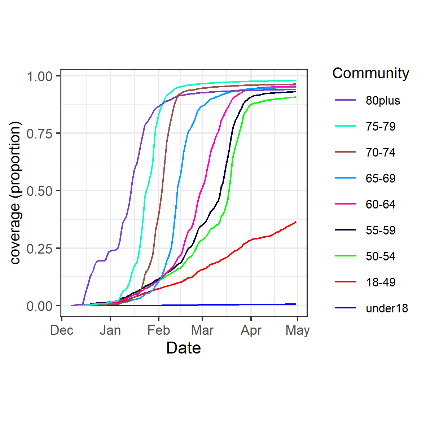 |

Figure 2: Mathematical model schematic and observed stratified data for Alpha variant cases and vaccination proportions (example)

A) Individuals can be in any of the infection states Susceptible, Exposed (infected but not yet infectious), Infected (infected and infectious, including non-symptomatic), or Recovered, and transition between states probabilistically. Patient-facing hospital HCW can be infected within the hospital by patients or other staff and by the general community while they are not on shift.

B) Proportion of new infections that are Alpha variant by PCR sample date stratified by NHSE region (Volz et al.^28^)

C) Proportion of hospital HCW received at least one dose of COVID-19 vaccine one or more doses of COVID-19 vaccine by first vaccine date stratified by GOR region (SIREN/NIMS)

D) Proportion of adults in England who received at least one dose of COVID-19 vaccine by first vaccine date stratified by age-band in JCVI prioritisation (NIMS)

## Model Calibration

Where data were available, we assigned values to identifiable parameters from the literature (Appendix Table A1). The unidentifiable parameters were iteratively calibrated to within acceptable ranges using the methodology described by Joslyn et al.^11^. We derived a set of criteria against which simulations were deemed to be representative of observed data using aggregated hospital-level data on the infection rate in patients and HCWs drawn; these criteria are defined in Table 2. Initially log-10 uniform probability distributions were assumed for all transmission probabilities and parameters were allowed to take on any value from 10 ^-10^ to 1. 1000 Latin Hypercube sampled parameter sets were generated and simulations were executed as described in “Simulations”. For the next iteration a new distribution was derived for each parameter set by identifying the highest-density regions for each parameter set in which any of the tests were satisfied. This was repeated for two further iterations until a subset of runs passed all four tests. The parameter sets that passed all four tests were then assessed qualitatively against empirical data on patient and HCW infections, so compared qualitatively to the parameter values to those in a previously published simple deterministic model of hospital transmission^12^.

The proportion of susceptible patients that acquired a nosocomial infection was estimated to be less than 5% based on a previous modelling study demonstrating this outcome in a high prevalence area with higher than normal transmission rates^12^. We used data from the SIREN study to estimate the proportion of HCWs infected over time. The range into which a result must fall to pass the test relating to each criterion is detailed in Table 2. The final fitted output following calibration is shown in Figure 3. It should be noted that from February 2022 community testing rates rapidly declined. This impacts the number of community-onset suspected hospital-associated cases identified in the data after this time and is responsible for the divergence of simulated estimates from national data.

Table 2: Criteria for an outcome to be defined as related to a feasible parameter set

| Criteria | | Min | Max |
| --- | --- | --- | --- |
| 1 | Maximum proportion of susceptible patients that develop a nosocomial infection | 0.0005 | 0.05 |
| 2 | Proportion of HCWs infected before 10 November 2020 | 0.202 | 0.353 |
| 3 | Proportion of HCWs infected before 08 December 2020 | 0.208 | 0.382 |
| 4 | Proportion of HCWs infected between 10 November and 08 December 2020 | 0.003 | 0.021 |

**A**


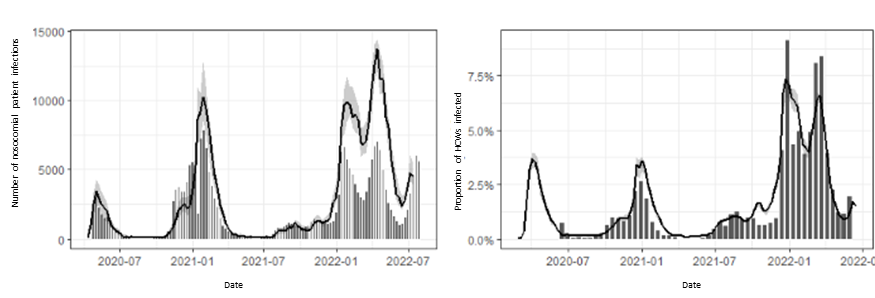


**B**

**Figure 3: Calibration of simulation output to data.** A) Simulation output (black line median, grey ribbon, IQR), for patient infections over time compared to data from the UKHSA unified infection dataset (bars) for the same measure in England. B) Calibration of simulation output for HCW infections infected over time compared to SIREN study data (bars) over the same period (Foulkes et al.^29^).

## Input Data

The model relies on input data in the form of a file containing the number of cases to admit per day, this file must be named “region.csv” where region is one of those defined in Table 1. The parameters are also imported from an XML parameter file, examples of these files are contained in the Appendix below.

## Computational platform

The model is constructed in the Java programming language using Java SE 11. The IBM is cross-platform and runs without visualisation software. Simulations were executed locally.

**Simulations**

The model was simulated for 5100 timesteps covering a time period of 850 days from 03-March-2020 to 30-June-2022 aligning with the collection period of the patient-level and SIREN data. Individual-level patient and HCW data on infection status and location were recorded at each time step.

## Scaling between regional and national data

To scale regional data up to a national level, the results are transformed into infections per bed and proportion of workforce infected and then multiplied by the number of beds available or total patient-facing HCW population nationally. ^13^.

**Submodels**

*Patient-HCW interaction model:* Patient and HCW interactions are randomly generated at each time step, with each HCW randomly selecting 3 beds to visit per time step. For ward-based HCWs these patients will be within the ward the HCW is working on, and for transient staff they can be anywhere in the hospital.

*Infectiousness model:* When individuals become infected a peak Ct-value, timestep-to-peak, and recovery time are drawn at random. The individual-level Ct-values are then calculated at every step (Figure 4). As described by Quilty et al [3], an individual is infectious when their Ct-levels are below 30.


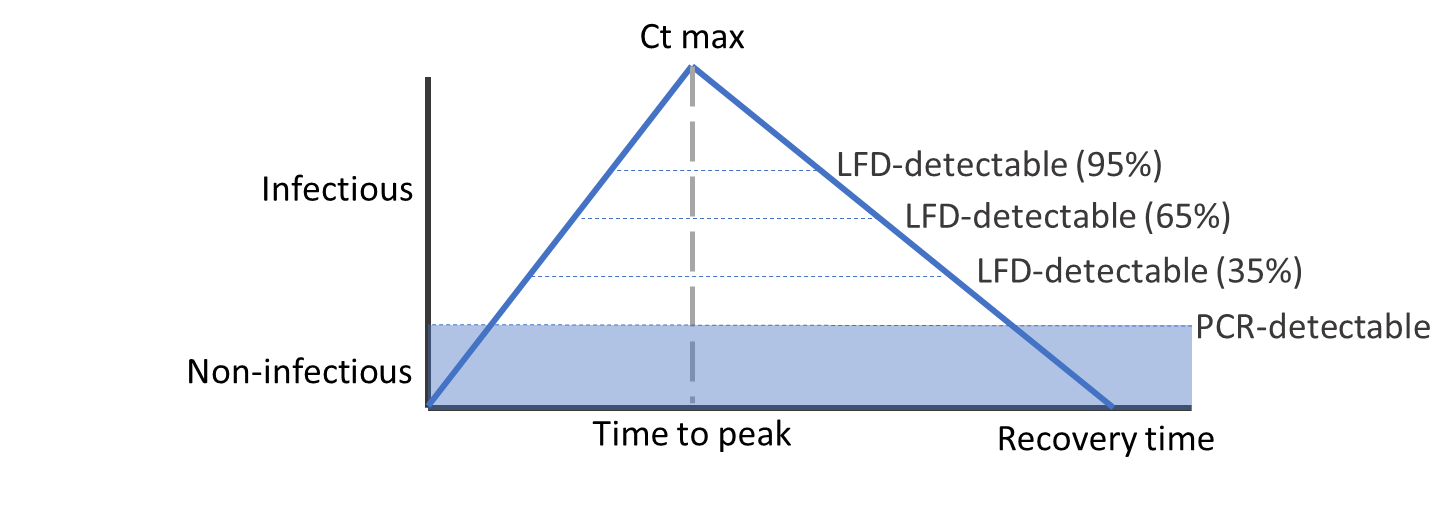


**Figure 4: Infectiousness model (adapted from Quilty et al [3]).**

*Vaccine uptake and effectiveness model:* Vaccine uptake is modelled by age group the probability of becoming vaccinated at any time step drawn from a logistic curve. Vaccine efficacy is initially modelled as a Heaviside function, but protection begins to wane after 28 days (Figure 5).


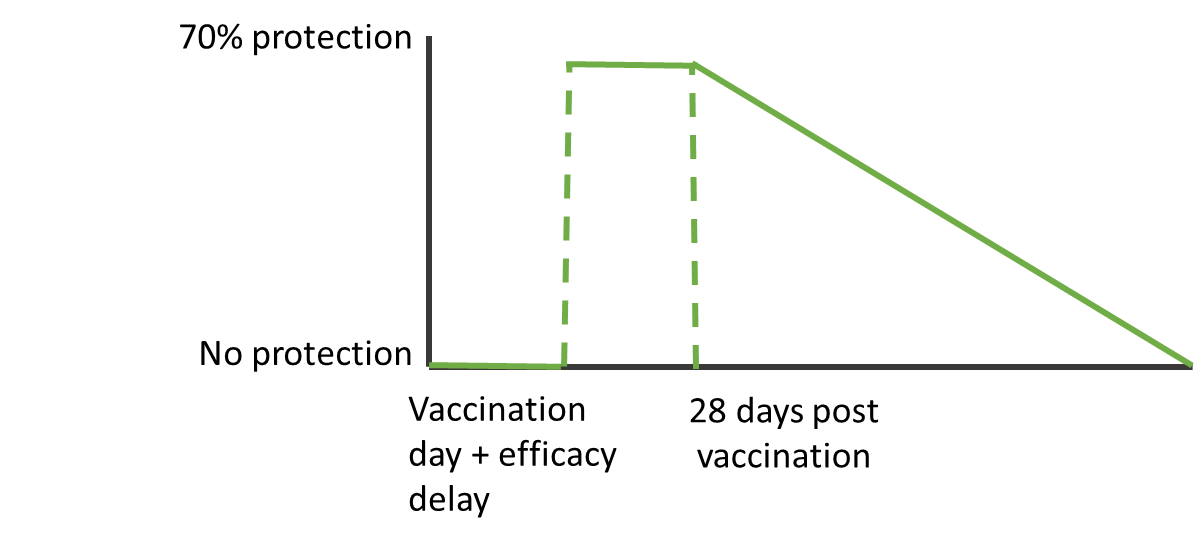


**Figure 5: Vaccine efficacy schematic**

# **References**

1. Grimm V, Berger U, DeAngelis DL, et al. The ODD protocol: A review and first update. *Ecological Modelling* 2010;221(23):2760-68. doi: <https://doi.org/10.1016/j.ecolmodel.2010.08.019>

2. Luke S, Cioffi-Revilla C, Panait L, Sullivan K, Balan G. Mason: A multiagent simulation environment. *Simulation* 2005;81(7):517-27.

3. Office for National Statistics. National population projections: 2018-based. Office for National Statistics. <https://www.ons.gov.uk/peoplepopulationandcommunity/populationandmigration/populationprojections/bulletins/nationalpopulationprojections/2018based>.

4. Joint Committee on Vaccination and Immunisation. Joint Committee on Vaccination and Immunisation: advice on priority groups for COVID-19 vaccination, 30 December 2020. 6 January 2021. <https://www.gov.uk/government/publications/priority-groups-for-coronavirus-covid-19-vaccination-advice-from-the-jcvi-30-december-2020/joint-committee-on-vaccination-and-immunisation-advice-on-priority-groups-for-covid-19-vaccination-30-december-2020>.

5. The Kings Fund. Overview of the health and social care workforce. <https://www.kingsfund.org.uk/projects/time-think-differently/trends-workforce-overview>.

6. Birrell P, Blake J, Van Leeuwen E, Gent N, De Angelis D. Real-time nowcasting and forecasting of COVID-19 dynamics in England: the first wave. *Philos Trans R Soc Lond B Biol Sci* 2021;376(1829):20200279. doi: 10.1098/rstb.2020.0279

7. Delignette-Muller ML, Dutang CJJoss. fitdistrplus: An R package for fitting distributions. 2015;64(4):1-34.

8. Bhattacharya A, Collin SM, Stimson J, et al. Healthcare-associated COVID-19 in England: a national data linkage study. *J Infect* 2021 doi: 10.1016/j.jinf.2021.08.039

9. Cohen J, Bilsen J, Addington-Hall J, et al. Population-based study of dying in hospital in six European countries. *Palliat Med* 2008;22(6):702-10. doi: 10.1177/0269216308092285

10. Evans S, Stimson J, Pople D, et al. Quantifying the contribution of pathways of nosocomial acquisition of COVID-19 in English hospitals. *Int J Epidemiol* 2021 doi: 10.1093/ije/dyab241

11. Joslyn LR, Kirschner DE, Linderman JJ. CaliPro: A Calibration Protocol That Utilizes Parameter Density Estimation to Explore Parameter Space and Calibrate Complex Biological Models. *Cell Mol Bioeng* 2021;14(1):31-47. doi: 10.1007/s12195-020-00650-z

12. Evans S, Agnew E, Vynnycky E, et al. The impact of testing and infection prevention and control strategies on within-hospital transmission dynamics of COVID-19 in English hospitals. *Philos Trans R Soc Lond B Biol Sci* 2021;376(1829):20200268. doi: 10.1098/rstb.2020.0268

13. NHS England. Bed Availability and Occupancy Data – Overnight. <https://www.england.nhs.uk/statistics/statistical-work-areas/bed-availability-and-occupancy/bed-data-overnight/>.

14. Nuffield Trust. Hospital bed occupancy. <https://www.nuffieldtrust.org.uk/resource/hospital-bed-occupancy>.

15. Woloshin S, Patel N, Kesselheim AS. False Negative Tests for SARS-CoV-2 Infection - Challenges and Implications. *N Engl J Med* 2020;383(6):e38. doi: 10.1056/NEJMp2015897

16. University of Oxford SARS-CoV-2 test development validation cell. Preliminary report from the Joint PHE Porton Down & University of Oxford SARS-CoV-2 test development and validation cell: Rapid evaluation of Lateral Flow Viral Antigen detection devices (LFDs) for mass community testing. <https://www.ox.ac.uk/sites/files/oxford/media_wysiwyg/UK%20evaluation_PHE%20Porton%20Down%20%20University%20of%20Oxford_final.pdf>.

17. He X, Lau EH, Wu P, et al. Temporal dynamics in viral shedding and transmissibility of COVID-19. *Nature medicine* 2020;26(5):672-75.

18. Office for National Statistics. Population estimates for the UK, England and Wales, Scotland and Northern Ireland: mid-2018. Office for National Statistics; 27 June 2019. <https://www.ons.gov.uk/peoplepopulationandcommunity/populationandmigration/populationestimates/bulletins/annualmidyearpopulationestimates/mid2018>.

19. Office for Health Improvement Disparities. AMR local indicators - OHID. <https://fingertips.phe.org.uk/amr-local-indicators#gid/1938132917/ati/118>.

20. Cohen B, Hyman S, Rosenberg L, Larson E. Frequency of patient contact with health care personnel and visitors: implications for infection prevention. *The Joint Commission Journal on Quality and Patient Safety* 2012;38(12):560-65.

21. Public Health England. UK flu levels according to PHE statistics: 2018 to 2019. <https://www.gov.uk/government/news/uk-flu-levels-according-to-phe-statistics-2018-to-2019>.

22. Kluytmans-van den Bergh MFQ, Buiting AGM, Pas SD, et al. Prevalence and Clinical Presentation of Health Care Workers With Symptoms of Coronavirus Disease 2019 in 2 Dutch Hospitals During an Early Phase of the Pandemic. *Jama Netw Open* 2020(2574-3805 (Electronic)) doi: doi: 10.1001/jamanetworkopen.2020.9673

23. NHS England, NHS Improvement. Novel coronavirus (COVID-19) standard operating procedure NHS England and NHS Improvement rollout of lateral flow devices for asymptomatic staff testing for SARS CoV-2 (phase 2: trusts). 16 November 2020 <https://www.england.nhs.uk/coronavirus/wp-content/uploads/sites/52/2020/11/C0873_i_SOP_LFD-rollout-for-asymptomatic-staff-testing_phase-2-trusts-v1.1_16-nov20.pdf>.

24. Jarvis C, Edmunds J, CMMID COVID-19 Working Group. Social contacts in workplace the UK from the CoMix social contact survey. 29 June 2021. <https://assets.publishing.service.gov.uk/government/uploads/system/uploads/attachment_data/file/1009160/S1311_Comix_Report_contacts_in_the_workplace_SAGE_93.pdf>.

25. Harris RJ, Hall JA, Zaidi A, et al. Effect of Vaccination on Household Transmission of SARS-CoV-2 in England. *N Engl J Med* 2021;385(8):759-60. doi: 10.1056/NEJMc2107717

26. Hall VJ, Foulkes S, Saei A, et al. COVID-19 vaccine coverage in health-care workers in England and effectiveness of BNT162b2 mRNA vaccine against infection (SIREN): a prospective, multicentre, cohort study. *Lancet* 2021;397(10286):1725-35. doi: 10.1016/s0140-6736(21)00790-x

27. Andrews N, Tessier E, Stowe J, et al. Vaccine effectiveness and duration of protection of Comirnaty, Vaxzevria and Spikevax against mild and severe COVID-19 in the UK. *Medrxiv* 2021

28. Volz E, Mishra S, Chand M, et al. Assessing transmissibility of SARS-CoV-2 lineage B.1.1.7 in England. *Nature* 2021;593(7858):266-69. doi: 10.1038/s41586-021-03470-x

29. Foulkes S, Monk EJM, Sparkes D, et al. Early Warning Surveillance for SARS-CoV-2 Omicron Variants, United Kingdom, November 2021-September 2022. *Emerg Infect Dis*. 2023 Jan;29(1):184-188. doi: 10.3201/eid2901.221293.

# Appendix : Parameter Value Tables and Example Input Files

Appendix Table A1: Parameter values

| **General** |  |  |  |
| --- | --- | --- | --- |
| occupancy | Proportion of occupied beds in trust | 0.85 | Mean from individual trust data in SUS |
| hcws | Number of HCWs | 8000 | Mean from individual trust data |
| patients | Number of patients initially in trust | 860 | At 86% capacity^14^ |
| testSens_PCR | Sensitivity of PCR test (false negative rate) | 0.95 | Woloshin et al.^15^ |
| testSpec_PCR | Specificity of PCR test (false positive rate) | 0.995 | Woloshin et al.^15^ |
| testSens_LFD | Sensitivity of LFD test (false negative rate) | 0.78 | PHE/University of Oxford^16^ |
| testSpec_LFD | Specificity of LFD test (false positive rate) | 0.995 | PHE/University of Oxford^16^ |
| incubationPeriodDays_shape | Shape variable for Gamma distribution for incubation period | 13.3 | He et al.^17^ |
| incubationPeriodDays_rate | Rate variable for Gamma distribution for incubation period | 4.16 | He et al.^17^ |
| onsetPeriodDays_mean | Mean for Log-normal distribution for onset period | 1.434065 | SPI-M communication |
| onsetPeriodDays_sd | SD for Log-normal distribution for onset period | 0.6612 | SPI-M communication |
| camPop | Community population (Cambridgeshire) | 651482 | 2018 mid-year estimate^18^ |
| wards | Number of wards | 42 |  |
| shared_bays | Number of bays | 4 | ~80% beds are in shared rooms^19^ |
| bedsPerBay | Number of beds in a shared room | 6 |  |
| **Patients** |  |  |  |
| probMale | Probability of patients being Male | 0.576 | SUS |
| ageShape | Shape component of Gamma distribution of age distribution | 4.145 | Fit to data from SUS |
| ageScale | Scale component of Gamma distribution of age distribution | 75.48 | Fit to data from SUS |
| shapeLOS_Susc | Shape parameter for LOS distribution Weibull distribution for susceptible patients | -0.1780082 | Fit to data from SUS |
| scaleLOS_Susc_int | Intercept for scale parameter for LOS distribution Weibull distribution for susceptible patients | 2.331949 | Fit to data from SUS |
| scaleLOS_Susc_age | Age component of scale parameter for LOS distribution Weibull distribution for susceptible patients | -0.00546131 | Fit to data from SUS |
| scaleLOS_Susc_female | Female gender component of scale parameter for LOS distribution Weibull distribution for susceptible patients | -1.242609 | Fit to data from SUS |
| scaleLOS_Susc_age_female | Interaction of age and female gender component of scale parameter for LOS distribution Weibull distribution for susceptible patients | 0.01424664 | Fit to data from SUS |
| shapeLOS_Inf | Shape parameter for LOS distribution Weibull distribution for symptomatically infected patients | 0.07853536 | Fit to data from SUS |
| scaleLOS_Inf_int | Intercept for scale parameter for LOS distribution Weibull distribution for symptomatically infected patients | 2.595361 | Fit to data from SUS |
| scaleLOS_Inf_age | Age component of scale parameter for LOS distribution Weibull distribution for susceptible patients | 0.002301093 | Fit to data from SUS |
| scaleLOS_Inf_female | Female gender component of scale parameter for LOS distribution Weibull distribution for susceptible patients | -0.5465226 | Fit to data from SUS |
| scaleLOS_Inf_age_female | Interaction of age and female gender component of scale parameter for LOS distribution Weibull distribution for susceptible patients | 0.006978023 | Fit to data from SUS |
| patientAsymProb | Probability a patient infection is asymptomatic | 0.4 | SUS |
| bP2P | Transmission rate to other patients | Varied between [0.028, 0.086] in the  parameter sets | Calibrated |
| bH2P | Transmission rate from HCW to patients | Varied between [0.00001, 0.00063]  in the parameter sets | Calibrated |
| bP2P_hosp | Indirect transmission rate from patients to other patients | Varied between [0.000013, 0.00069]  in the parameter sets | Calibrated |
| recoveryRatePat_Shape | Shape parameter of Gamma distribution of recovery distribution per day. | 1.43 | Fit to data from SUS |
| recoveryRatePat_Rate | Rate parameter of Gamma distribution of recovery distribution per day. | 0.542 | Fit to data from SUS |
| deathOnDisch_Inf_Int | Intercept of linear model for probability of death on discharge for infected patients | -0.00043572 | Fit to data from SUS |
| deathOnDisch_Inf_X | X component of linear model for probability of death on discharge for infected patients | -0.00159802 | Fit to data from SUS |
| deathOnDisch_Inf_X2 | X^2^ component of linear model for probability of death on discharge for infected patients | 7.30529E-05 | Fit to data from SUS |
| deathOnDisch_Susc_Int | Intercept of linear model for probability of death on discharge for non-symptomatic/uninfected patients | -7.96613 | Fit to data from Cohen et al.^9^ |
| deathOnDisch_Susc_X | X component of linear model for probability of death on discharge for non-symptomatic/uninfected patients | 0.00102 | Fit to data from Cohen et al.^9^ |
| deathOnDisch_Susc_X2 | X^2^ component of linear model for probability of death on discharge for non-symptomatic/uninfected patients | 0.000896 | Fit to data from Cohen et al.^9^ |
| testOnAdmProb_Inf | Probability a symptomatically infected patient will be tested on admission | 0.95 | Estimated from individual NHSE trust data |
| testOnAdmProb_Other | Probability non-infected patient will be tested at random on admission | 0.05 | Estimated from individual NHSE trust data |
| testInHospProb | Probability a symptomatically infected patient infected nosocomially will be tested per timestep | 0.1 | Assumption that all patients will be tested within 2 days of developing symptoms |
| testPeriodSteps | Timesteps from test to result | 8 | Estimated from individual NHSE trust data |
| readmitProb | Probability a patient that develops symptoms after discharge will be readmitted within 14 days | 0.2 | Estimated from individual NHSE trust data |
| expOnAdmissionProb | Probability a susceptible patient will be exposed on admission (to be multiplied by the number of cases admitted that are known symptomatic) | 0.002631579 |  |
| retestProb_per_step | Probability a patient will be retested after day 5 (per step) | 0 | No retesting in this analysis |
| hcw_to_patient_contacts | Number of unique patients seen by a HCW every day (used to calculate exposure risk to COVID+ patients) | 20 | Cohen et al.^20^ |
| allAdmTestStartDay | Day to start testing all admissions | 0 | Assume all admissions are always tested/retested |
| d3TestStartDay | Day to start retesting on day 3 | 0 |  |
| d5TestStartDay | Day to start retesting on day5 | 0 |  |
| non_covid_sympt_prob | Probability a non-infected patient with have COVID-19-like symptoms on admission | 0.1 | Assumption based on ILI proportions in a normal year^21^ |
| d0_exp_prob | Proportion of new admissions that have previously recovered from COVID-19 | 0.5 | SUS |
| visitorsPerStep | Probability of having a visitor in a single time step | Varied | 0 = no visitors, 0.021 = average of 3 visitors a day from expert elicitation exercise. |
| d0_exp_prob_omicron | Proportion of new admissions that have previously recovered from omicron | 0.05 | SUS |
| **HCWs** |  |  |  |
| shiftLengthHrs | Length of shift in hours (this is converted to timesteps within the model code) | 12 | Assumption |
| hcwAsymProb | Probability a HCW that becomes infected is asymptomatic | 0.4 | Assumption |
| bP2H | Transmission probability from patients to HCWs per timestep | 0.0000025 | Calibrated |
| bH2H | Transmission probability from HCWs to other HCWs per timestep | 0.0000001 | Calibrated |
| absentThroughSick_self | Probability a HCW will self-isolate per timestep | 0.01 | 34% over total infected time^22^ |
| testProbHCWDays | Periodicity of HCW LFD testing (days) | 3.5 | NHSE LFD protocol^23^ |
| lfd_test_compliance | Proportion of HCWs adhering to LFD testing regime | 0.7 | Expert opinion |
| absentDays | Number of days to be off work following a positive test | 7 |  |
| beginTestingDay | Day that LFD testing of HCW began | 253 |  |
| commScale | Scale of community acquisition rate for HCWs | 0.052 | CoMIX^24^ |
| patient_to_hcw_contacts | Number of HCWs seen by a patient per day (for scaling transmission risk) | 18 | Cohen et al.^20^ |
| prob_ward_based | Probability a HCW is based on a ward instead of moving around the hospital | 0.25 | Estimate from proportion of nursing staff out of all staff |
| days_to_vaccine_efficacy | Day after vaccination that protection begins | 21 |  |
| Vaccine_waning_per_step | Rate of vaccine waning per time step | 0.003 | Andrews et al [6] |
| Vaccine_efficacy_one_dose | Efficacy of one vaccine dose | 0.7 | SIREN study estimates |
| Vaccine_efficacy_two_doses | Efficacy of two vaccine doses | 0.8 | UKHSA technical briefing |
| Vaccine_efficacy_one_dose_omicron | Efficacy of one vaccine doses against omicron | 0 |  |
| Vaccine_efficacy_two_doses_omicron | Efficacy of two vaccine doses against omicron | 0.32 |  |
| Vaccine_efficacy_three_doses_omicron | Efficacy of three vaccine doses against omicron | 0.62 |  |
| Vaccine_efficacy_no_doses_omicron_prior | Efficacy of protection against omicron in those that have prior infection | 0.44 |  |
| Vaccine_efficacy_one_dose_omicron_prior | Efficacy of one vaccine doses against omicron in those that have prior infection | 0.44 |  |
| Vaccine_efficacy_two_doses_omicron_prior | Efficacy of two vaccine doses against omicron in those that have prior infection | 0.6 |  |
| Vaccine_efficacy_three_doses_omicron_prior | Efficacy of three vaccine doses against omicron in those that have prior infection in those that have prior infection | 0.71 |  |
| hcw_vaccination_start_day | Day that vaccination program in HCWs commences | 281 | 8 December 2021 |
| **Comm** |  |  |  |
| careHomeProb | Probability a patient is from a care home | 0.02 | Health foundation communication |
| caseScale | Scaling factor for admissions rate | 1 |  |
| vacc_scale_from | Scaling factor for transmissibility from vaccinated individual | 0.5 | Harris et al.^25^ |
| vacc_scale_to1 | Scaling factor for transmissibility to individual vaccinated with one dose (trans = 1-value) | 0.7 | Hall et al.^26^ |
| vacc_scale_to2 | Scaling factor for transmissibility to individual vaccinated with two doses (trans = 1-value) | 0.7 | Andrews et al.^27^ |
| var_scale_alpha | Scaling factor of transmission parameter for Alpha variant (compared to WT) | 1.56 | PHE technical report |
| var_scale_delta | Scaling factor of transmission parameter for Delta variant (compared to WT) | 1.99 | PHE technical report |
| var_scale_omicron | Scaling factor of transmission parameter for Omicron variant (compared to WT) | 2.32 | https://www.medrxiv.org/content/10.1101/2021.12.31.21268583v1.full |

Appendix Table A2: Logistic distribution coefficients for Alpha variant proportion

| Fit to period 1^st^ October – 22^nd^ January inclusive (day 0 = 1 March 2020)  Fitted function : $y = A/\left( 1+ exp(-k(t - \mu)) \right)$  t = week number, y = coverage proportion | | | | |
| --- | --- | --- | --- | --- |
|  | Fitted coefficients | | | Observed data |
| NHSE Region | A | k | μ | Source |
| London | 0.977 | 0.699 | 49.075 | Volz et al.^28^ |
| Midlands | 0.907 | 0.849 | 51.930 |  |
| North East and Yorkshire | 0.842 | 0.855 | 52.661 |  |
| North West | 0.911 | 0.750 | 52.74 |  |
| South East | 0.915 | 0.688 | 47.480 |  |
| South West | 0.957 | 0.610 | 51.666 |  |
| East of England | 0.951 | 0.713 | 49.291 |  |

Fitted using R package ‘drc’

Appendix Table A3: logistic distribution coefficients for vaccine cover proportion (1+ doses)

| Fit to period 7^th^ December – 9^th^ April inclusive, assuming day 0 (7^th^ December) has 0% coverage  Fitted function : $y = A/\left( 1+ exp(-k(t - \mu)) \right)$  t = day number, y = coverage proportion | | | | | |
| --- | --- | --- | --- | --- | --- |
|  |  | Fitted coefficients | | | Observed data |
|  |  | A | k | μ | Source |
| HCW | England | 0.931251619 | 0.116446417 | 31.90180448 | SIREN-NIMS |
| Population | 16-49 | 0.361368 | 0.0439 | 92.47233 | NIMS, ONS^3^ |
|  | 50-54 | 1.643953 | 0.049632 | 114.9436 |  |
|  | 55-59 | 1.257394 | 0.058007 | 99.44187 |  |
|  | 60-64 | 1.011423 | 0.089646 | 82.83167 |  |
|  | 65-69 | 1.011423 | 0.089646 | 82.83167 |  |
|  | 70-74 | 0.954113 | 0.227902 | 57.10629 |  |
|  | 75-79 | 0.979244 | 0.204977 | 48.48741 |  |
|  | 80plus | 0.955866 | 0.109699 | 35.95469 |  |

Input File Example 1: XML parameter file

| <?xml version="1.0" encoding="UTF-8" standalone="no"?>  <input>  <General>  <occupancy>0.85</occupancy>  <doctors>1600</doctors>  <nurses>6400</nurses>  <hcws>8000</hcws>  <patients>200</patients>  <recruitPatInt>-5.062</recruitPatInt>  <recruitPatX> 1.478</recruitPatX>  <recruitPatX2>-0.03795</recruitPatX2>  <recruitPatX3>0.0002646</recruitPatX3>  <testSens>0.95</testSens>  <testSpec>0.95</testSpec>  <incubationPeriodDays_shape>13.3</incubationPeriodDays_shape>  <incubationPeriodDays_rate>4.16</incubationPeriodDays_rate>  <onsetPeriodDays_mean>1.434065</onsetPeriodDays_mean>  <onsetPeriodDays_sd>0.6612</onsetPeriodDays_sd>  <camPop>651482</camPop>  <recScale>0.430980337293819</recScale>  <asympCommScale>233.039498227648</asympCommScale>  <wards>42</wards>  <single_bays>0</single_bays>  <shared_bays>4</shared_bays>  <bedsPerBay>6</bedsPerBay>  </General>  <Patients>  <probMale>0.576</probMale>  <ageShape>4.145</ageShape>  <ageScale>75.48</ageScale>  <shapeLOS_Susc>-0.17800824</shapeLOS_Susc>  <scaleLOS_Susc_int>2.33194900</scaleLOS_Susc_int>  <scaleLOS_Susc_age>-0.00546131</scaleLOS_Susc_age>  <scaleLOS_Susc_female>-1.24260868</scaleLOS_Susc_female>  <scaleLOS_Susc_age_female>0.01424664</scaleLOS_Susc_age_female>  <shapeLOS_Inf>0.078535365</shapeLOS_Inf>  <scaleLOS_Inf_int>2.595360634</scaleLOS_Inf_int>  <scaleLOS_Inf_age>0.002301093</scaleLOS_Inf_age>  <scaleLOS_Inf_female>-0.546522561</scaleLOS_Inf_female>  <scaleLOS_Inf_age_female>0.006978023</scaleLOS_Inf_age_female>  <patientAsymProb>0.4</patientAsymProb>  <bP2P>0.0022</bP2P>  <bP2P_hosp>0.000015</bP2P_hosp>  <bH2P>5.00E-04</bH2P>  <recoveryRatePat_Shape>1.43</recoveryRatePat_Shape>  <recoveryRatePat_Rate>0.542</recoveryRatePat_Rate>  <deathOnDisch_Inf_Int>-0.0004357198</deathOnDisch_Inf_Int>  <deathOnDisch_Inf_X>-0.0015980156</deathOnDisch_Inf_X>  <deathOnDisch_Inf_X2>0.0000730529</deathOnDisch_Inf_X2>  <deathOnDisch_Susc_Int>-7.96613</deathOnDisch_Susc_Int>  <deathOnDisch_Susc_X>0.00102</deathOnDisch_Susc_X>  <deathOnDisch_Susc_X2>0.000896</deathOnDisch_Susc_X2>  <testOnAdmProb_Inf>1</testOnAdmProb_Inf>  <testOnAdmProb_Other>1</testOnAdmProb_Other>  <testInHospProb>0.2</testInHospProb>  <testPeriodSteps>8</testPeriodSteps>  <readmitProb>0.2</readmitProb>  <expOnAdmissionProb>0.002631579</expOnAdmissionProb>  <testPosScale>0.0</testPosScale>  <testNegScale>1.0</testNegScale>  <suspectedScale>1.0</suspectedScale>  <retestProb_per_step>0.0</retestProb_per_step>  <dayToRetest>10000</dayToRetest>  <non_covid_sympt_prob>0.1</non_covid_sympt_prob>  <hcw_to_patient_contacts>20</hcw_to_patient_contacts>  </Patients>  <HCWs>  <shiftLengthHrs>12</shiftLengthHrs>  <hcwAsymProb>0.4</hcwAsymProb>  <bP2H>2.5E-04</bP2H>  <bH2H>1.5E-04</bH2H>  <absentThroughSick_self>0.1</absentThroughSick_self>  <absentThroughSick_random>0.005</absentThroughSick_random>  <testProbHCWDays>5000</testProbHCWDays>  <absentDays>10</absentDays>  <beginTestingDay>1</beginTestingDay>  <commScale>0.2</commScale>  <patient_to_hcw_contacts>18</patient_to_hcw_contacts>  <prob_ward_based>0.25</prob_ward_based>  <hcw_vaccination_start_day>281</hcw_vaccination_start_day>  <days_to_vaccine_efficacy>21</days_to_vaccine_efficacy>  <hcw_vaccine_eq_A>0.93</hcw_vaccine_eq_A>  <hcw_vaccine_eq_k>0.116446417</hcw_vaccine_eq_k>  <hcw_vaccine_eq_mu>31.90180448</hcw_vaccine_eq_mu>  <vaccine_efficacy_one_dose>0.76</vaccine_efficacy_one_dose>  </HCWs>  <Comm>  <commProbInt>-0.00000469835</commProbInt>  <commProbX> 0.000001895326</commProbX>  <commProbX2>-0.00000002551533</commProbX2>  <minCommProb>0.0000015</minCommProb>  <symptomToHospDays>0</symptomToHospDays>  <careHomeProb>0.1</careHomeProb>  <caseScale>1</caseScale>  <visitorsPerStep>0</visitorsPerStep>  <extraLOS>1.42</extraLOS>  <prop_117_eq_A>1.0</prop_117_eq_A>  <prop_117_eq_k>0.63</prop_117_eq_k>  <prop_117_eq_mu>57.4</prop_117_eq_mu>  <vacc_scale_from>0.5</vacc_scale_from>  <var_scale_from>1.57</var_scale_from>  <comm_vac_16_to_49_A>0.361</comm_vac_16_to_49_A>  <comm_vac_50_to_54_A>1.643</comm_vac_50_to_54_A>  <comm_vac_55_to_59_A>1.257</comm_vac_55_to_59_A>  <comm_vac_60_to_64_A>1.011</comm_vac_60_to_64_A>  <comm_vac_65_to_69_A>0.928</comm_vac_65_to_69_A>  <comm_vac_70_to_74_A>0.954</comm_vac_70_to_74_A>  <comm_vac_75_to_79_A>0.979</comm_vac_75_to_79_A>  <comm_vac_80_plus_A>0.955</comm_vac_80_plus_A>  <comm_vac_16_to_49_k>0.043</comm_vac_16_to_49_k>  <comm_vac_50_to_54_k>0.049</comm_vac_50_to_54_k>  <comm_vac_55_to_59_k>0.058</comm_vac_55_to_59_k>  <comm_vac_60_to_64_k>0.089</comm_vac_60_to_64_k>  <comm_vac_65_to_69_k>0.181</comm_vac_65_to_69_k>  <comm_vac_70_to_74_k>0.227</comm_vac_70_to_74_k>  <comm_vac_75_to_79_k>0.204</comm_vac_75_to_79_k>  <comm_vac_80_plus_k>0.109</comm_vac_80_plus_k>  <comm_vac_16_to_49_mu>92.472</comm_vac_16_to_49_mu>  <comm_vac_50_to_54_mu>114.943</comm_vac_50_to_54_mu>  <comm_vac_55_to_59_mu>99.441</comm_vac_55_to_59_mu>  <comm_vac_60_to_64_mu>82.831</comm_vac_60_to_64_mu>  <comm_vac_65_to_69_mu>68.039</comm_vac_65_to_69_mu>  <comm_vac_70_to_74_mu>57.106</comm_vac_70_to_74_mu>  <comm_vac_75_to_79_mu>48.487</comm_vac_75_to_79_mu>  <comm_vac_80_plus_mu>35.954</comm_vac_80_plus_mu>  </Comm>  </input> |
| --- |

Input File Example 2: Admissions File

| \| X \| y \| region \| \| --- \| --- \| --- \| \| 0 \| 0 \| East Of England \| \| 1 \| 0 \| East Of England \| \| 2 \| 0 \| East Of England \| \| 3 \| 0 \| East Of England \| \| 4 \| 0 \| East Of England \| \| 5 \| 0 \| East Of England \| \| 6 \| 0 \| East Of England \| \| 7 \| 0 \| East Of England \| \| 8 \| 0 \| East Of England \| \| 9 \| 0 \| East Of England \| \| 10 \| 0.000336 \| East Of England \| \| 11 \| 0.001532 \| East Of England \| \| 12 \| 0.002727 \| East Of England \| \| 13 \| 0.003923 \| East Of England \| \| 14 \| 0.005119 \| East Of England \| \| 15 \| 0.006315 \| East Of England \| \| 16 \| 0.007508 \| East Of England \| \| 17 \| 0.008691 \| East Of England \| \| 18 \| 0.009854 \| East Of England \| \| 19 \| 0.010982 \| East Of England \| \| 20 \| 0.012061 \| East Of England \| |
| --- | --- | --- | --- | --- | --- | --- | --- | --- | --- | --- | --- | --- | --- | --- | --- | --- | --- | --- | --- | --- | --- | --- | --- | --- | --- | --- | --- | --- | --- | --- | --- | --- | --- | --- | --- | --- | --- | --- | --- | --- | --- | --- | --- | --- | --- | --- | --- | --- | --- | --- | --- | --- | --- | --- | --- | --- | --- | --- | --- | --- | --- | --- | --- | --- | --- | --- |
