# Supplementary material for: Impact of interventions to reduce nosocomial transmission of SARS-CoV-2 in English NHS Trusts: a computational modelling study
Source: BMC Infect Dis. 2024 May 7;24:475. doi: 10.1186/s12879-024-09330-z (PMC11075183; doi:10.1186/s12879-024-09330-z)
Supplement: Supplementary file 2 [file 12879_2024_9330_MOESM2_ESM.pdf]

Supplementary File 2

Expert elicitation

Expert elicitations are a way of providing ‘best estimates’ for parameter values when there is a paucity of evidence in the literature. Our elicitation process involved contacting multiple experts and asking them to answer six multipart questions using a ‘chips and bins’ approach. This approach results in distributions that can be used to form ranges for the parameters of interest. In our study experts were asked questions about the efficacy and compliance with interventions to reduce the spread of SARS-CoV-2 in hospitals and given 20 ‘chips’ to be placed in ‘bins’ at 5% intervals. The elicitation exercise was conducted in January 2022. Response distributions were created in *R* using the density of each individual respondent. Where possible, elicited ranges were used to supplement data from the literature to generate parameter ranges that adequately reflect reality. Participants were provided with clear instructions and an introductory example before being asked to complete the questions of interest. A sample of the worksheet sent to participants is included in Supplementary File 3.

The corresponding distributions are as follows;

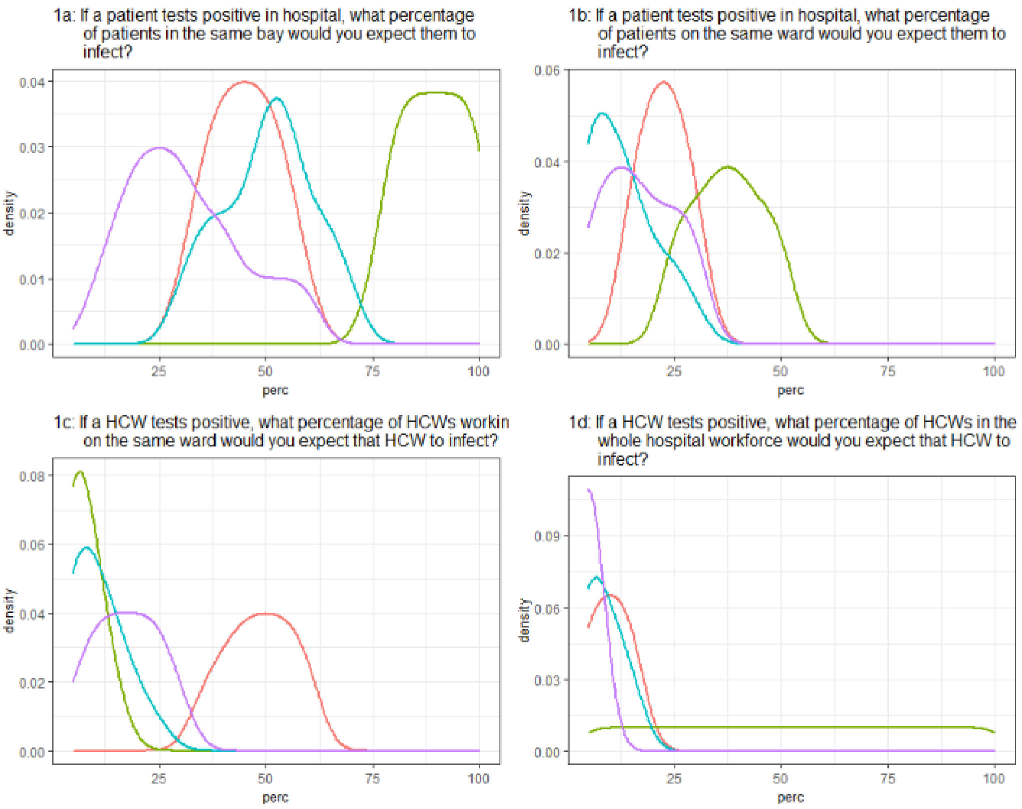

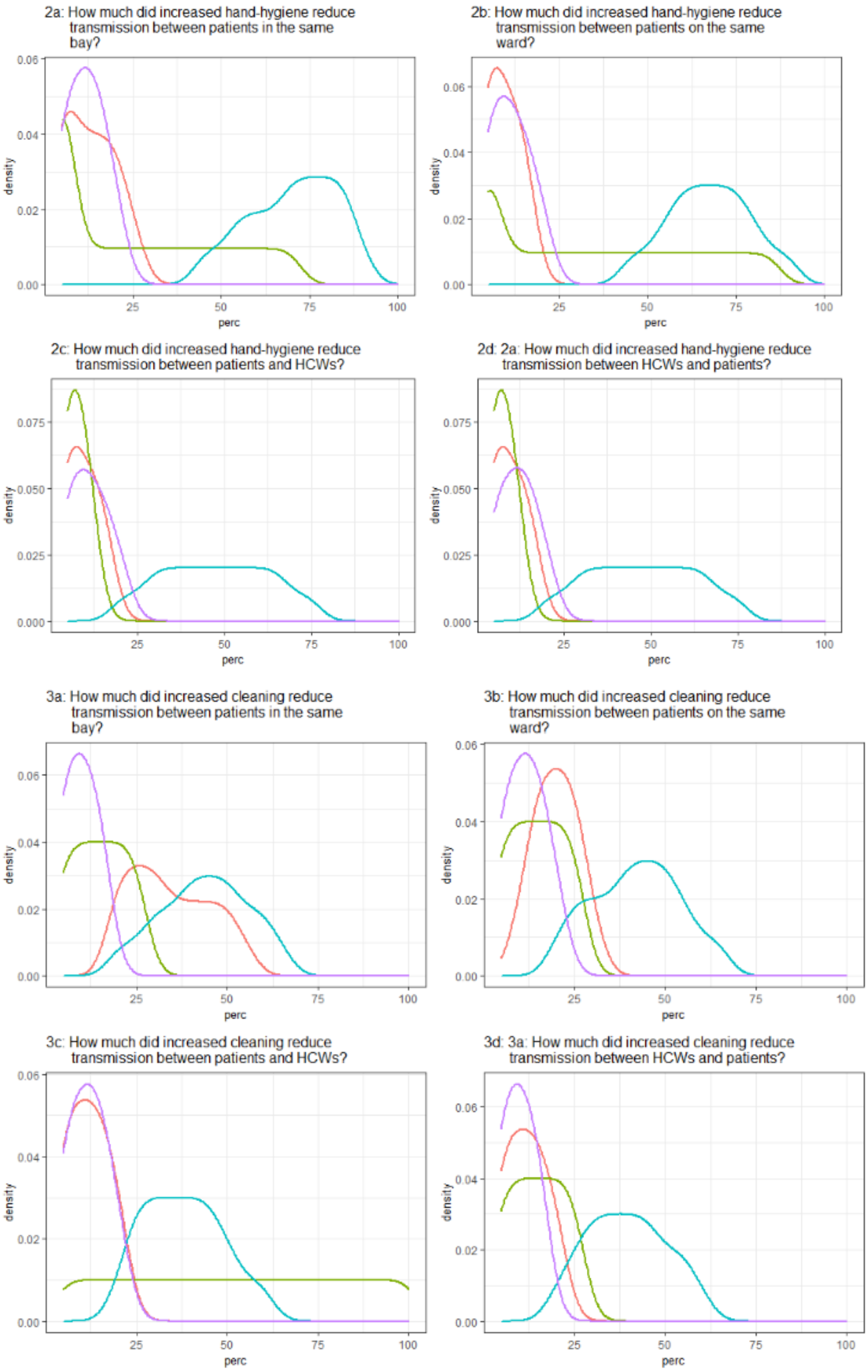
2a: How much did increased hand-hygiene reduce transmission between patients in the same bay?
2b: How much did increased hand-hygiene reduce transmission between patients on the same ward?
2c: How much did increased hand-hygiene reduce transmission between patients and HCWs?
2d: 2a: How much did increased hand-hygiene reduce transmission between HCWs and patients?
3a: How much did increased cleaning reduce transmission between patients in the same bay?
3b: How much did increased cleaning reduce transmission between patients on the same ward?
3c: How much did increased cleaning reduce transmission between patients and HCWs?
3d: 3a: How much did increased cleaning reduce transmission between HCWs and patients?
density
perc
25
50
75
100

4a: How much did universal masking by HCWs reduce transmission from patients to HCWs?

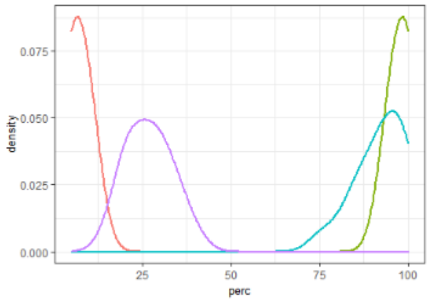

4b: How much did universal masking by HCWs reduce transmission from HCWs to patients?

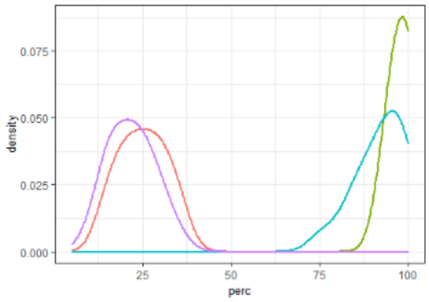

4c: How much did universal masking by HCWs reduce transmission between HCWs?

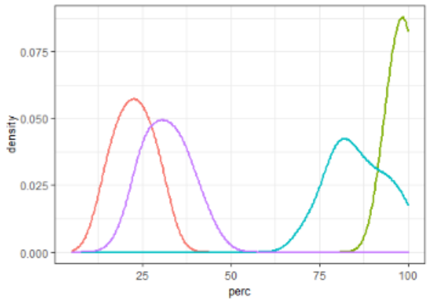

5a: What percent of their time while in the hospital do HCWs remain masked with patients?

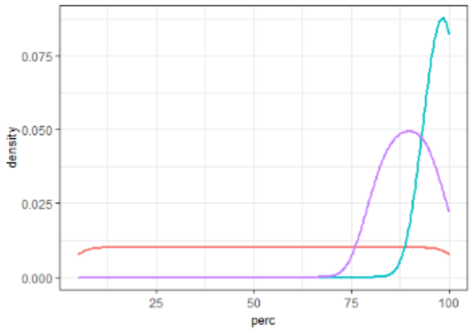

5b: What percent of their time while in the hospital do HCWs remain masked with other HCWs?

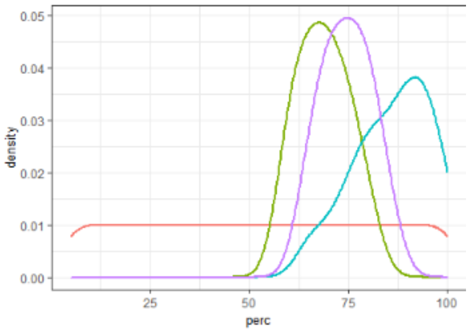

6a: How many non-HCWs visitors to a patient ward (e.g. for repairs or other people that are critical for service) were there in a day over the second wave?

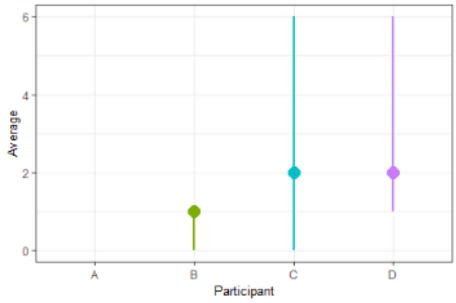

6b: How many non-HCWs visitors to a patient ward (e.g. for repairs or other people that are critical for service) were there in a day before the pandemic?

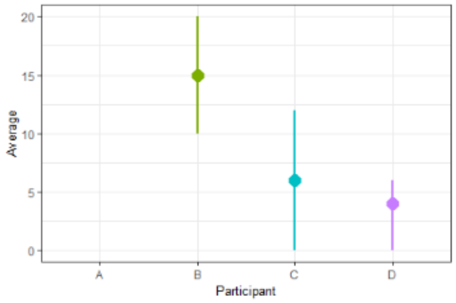

6c: How many personal visitors did an average patient get in a day before the pandemic?

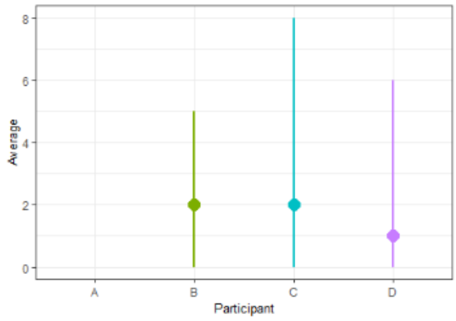

Figure 1. Distributions from expert elicitation. Responses from 4 experts shown. Straight horizontal lines indicate that expert did not feel confident assigning a distribution for a question.

Meta-analysis of efficacy of masking for reducing transmission to patients and HCWs

The efficacy of masks for preventing transmission of SARS-CoV-2 in hospitals was assessed though a meta-analysis. Twelve studies examining the effect of masks for reducing transmission of SARS-CoV-2 were analysed and included in this study [1–12]. Using the *meta* and *metafor R* packages, the number of cases and controls in these studies were included in the meta-analysis and a forest plot was generated. The estimate for the risk-ratio of those who wore masks compared to those who did not was 0.51 [0.28-0.95].

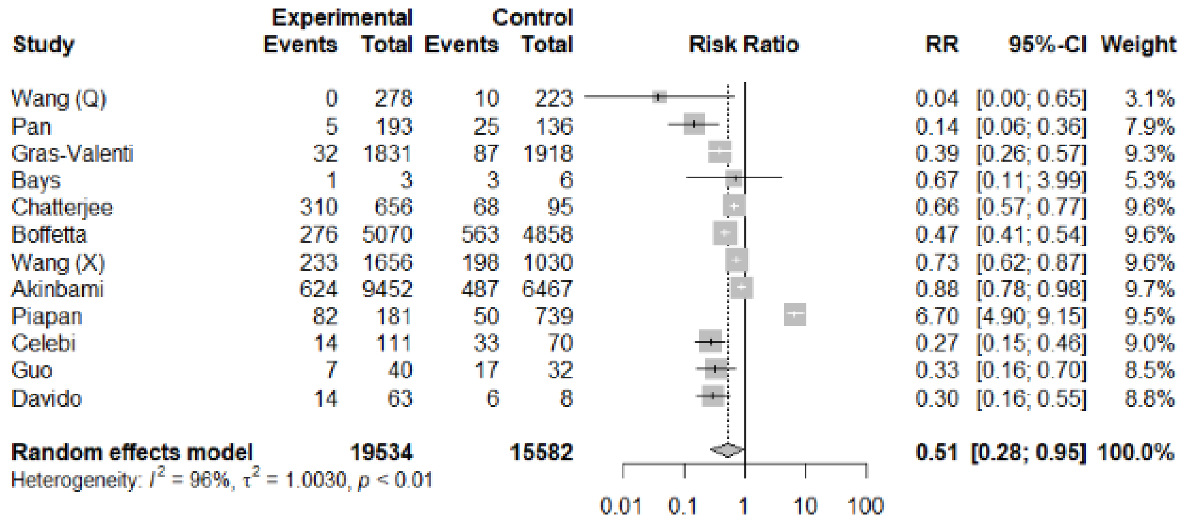

Figure 2: Forest plot of meta-analysis on studies of SARS-CoV-2 infection rates in HCWs that did or did not wear masks.

1. Wang X, Pan Z, Cheng Z. Association between 2019-nCoV transmission and N95 respirator use. Journal of Hospital Infection [Internet]. 2020 Mar 3 [cited 2020 Apr 3];0(0). Available from: https://www.journalofhospitalinfection.com/article/S0195-6701(20)30097-9/abstract

2. Gras-Valentí P, Mora-Muriel JG, Chico-Sánchez P, Algado-Sellés N, Soler-Molina VM, Hernández-Maldonado M, et al. Effectivity of a Program for the Control and Prevention of COVID-19 Healthcare-Associated Infections in a Spanish Academic Hospital. J Patient Saf. 2021 Jun;17(4):323–30.

3. Chatterjee P, Anand T, Singh KhJ, Rasaily R, Singh R, Das S, et al. Healthcare workers & SARS-CoV-2 infection in India: A case-control investigation in the time of COVID-19. Indian J Med Res. 2020 May;151(5):459–67.

4. Boffetta P, Violante F, Durando P, De Palma G, Pira E, Vimercati L, et al. Determinants of SARS-CoV-2 infection in Italian healthcare workers: a multicenter study. Sci Rep [Internet]. 2021 Mar

11 [cited 2022 Nov 30];11. Available from: https://www.ncbi.nlm.nih.gov/pmc/articles/PMC7970984/

5. Wang D. Clinical Characteristics of 138 Hospitalized Patients With 2019 Novel Coronavirus-Infected Pneumonia in Wuhan, China. - PubMed - NCBI [Internet]. 2020 [cited 2020 Feb 17]. Available from: https://www.ncbi.nlm.nih.gov/pubmed/?term=Clinical+Characteristics+of+138+Hospitalized+Patients+With+2019+Novel+Coronavirus%E2%80%93Infected+Pneumonia+in+Wuhan%2C+China

6. Piapan L, De Michieli P, Ronchese F, Rui F, Mauro M, Peresson M, et al. COVID-19 outbreak in healthcare workers in hospitals in Trieste, North-east Italy. J Hosp Infect. 2020 Nov;106(3):626–8.

7. Davido B, Gautier S, Riom I, Landowski S, Lawrence C, Thiebaut A, et al. The first wave of COVID-19 in hospital staff members of a tertiary care hospital in the greater Paris area: A surveillance and risk factors study. Int J Infect Dis. 2021 Apr;105:172–9.

8. Çelebi G, Pişkin N, Çelik Bekleviç A, Altunay Y, Salcı Keleş A, Tüz MA, et al. Specific risk factors for SARS-CoV-2 transmission among health care workers in a university hospital. Am J Infect Control. 2020 Oct;48(10):1225–30.

9. Bays DJ, Nguyen M-VH, Cohen SH, Waldman S, Martin CS, Thompson GR, et al. Investigation of nosocomial SARS-CoV-2 transmission from two patients to healthcare workers identifies close contact but not airborne transmission events. Infect Control Hosp Epidemiol. 2021 Sep;42(9):1046–52.

10. Akinbami LJ, Vuong N, Petersen LR, Sami S, Patel A, Lukacs SL, et al. SARS-CoV-2 Seroprevalence among Healthcare, First Response, and Public Safety Personnel, Detroit Metropolitan Area, Michigan, USA, May-June 2020. Emerg Infect Dis. 2020 Dec;26(12):2863–71.

11. Guo X, Wang J, Hu D, Wu L, Gu L, Wang Y, et al. Survey of COVID-19 Disease Among Orthopaedic Surgeons in Wuhan, People's Republic of China. J Bone Joint Surg Am [Internet]. 2020 Apr 23 [cited 2022 Nov 30]; Available from: https://www.ncbi.nlm.nih.gov/pmc/articles/PMC7188039/

12. Pan Z, Zhang H, Yang J, Tang S, Cheng Z, Wu K, et al. Surgical Masks for Protection of Health Care Personnel Against Covid-19: Results from an Observational Study. Clin Invest Med. 2021 Jun 14;44(2):E48-54.
